# Supplementary material for: PARP1 recruits DNA translocases to restrain DNA replication and facilitate DNA repair
Source: PLoS Genet. 2022 Dec 13;18(12):e1010545. doi: 10.1371/journal.pgen.1010545 (PMC9794062; doi:10.1371/journal.pgen.1010545)
Supplement: S1 Text — (PDF) [file pgen.1010545.s001.pdf]

PARP1 recruits DNA translocases to restrain DNA replication and facilitate DNA repair

Yen-Chih Ho, Chen-Syun Ku, Siang-Sheng Tsai, Jia-Lin Shiu, Yi-Zhen Jiang, Hui Emmanuela Miriam, Han-Wen Zhang, Yen-Tzu Chen, Wen-Tai Chiu, Song-Bin Chang, Che-Hung Shen, Kyungjae Myung, Peter Chi, Hungjiun Liaw

Supplementary Materials and Methods

The generation of gene-knockout T24 cells

PARP1-KO, HLTF-KO and ZRANB3-KO T24 cells were generated using the CRISPR/Cas9 gene knockout strategy by using a pLAS2w-lentiCRISPR (P2A) plasmid. Single-guide RNAs (sgRNA) targeting PARP1, HLTF and ZRANB3 were designed using the online tool CRISPR DESIGN (http://crispr.mit.edu/). The sgRNA sequence targeting PARP1 was 5'-GAG TCG AGT ACG CCA AGA GC-3', the sgRNA sequence targeting HLTF was 5'-GGT TGG ACT ACG CTA TTA CA-3', and the sgRNA sequence targeting ZRANB3 was 5'-GAA GGG ACC ACT ATT AAC AG-3'. The sgRNA fragments were ligated into the BsmBI sites of the pLAS2w-lentiCRISPR (P2A) plasmid. The resulting pLAS2w-lentiCRISPR plasmids were transfected into HEK293T cells using TransIT-LT1 Reagent (Mirus) to generate lentiviruses. T24 cells were then infected with the lentivirus and selected with 1 μg/ml puromycin for 10 days. Approximately 20 colonies from each knockout cell line were selected for western blotting analysis. The deletion of PARP1, HLTF and ZRANB3 was also confirmed by genomic sequencing.

RNA interference

Lentivirus-packaged shRNA was generated by the transfection of HEK293T cells with packing plasmids, pCMVΔR8.91 and pMD.G, and a pLKO_TRC005 vector containing control shLacZ, shSHPRH, shSMARCAL1, or shPARP1. The shRNA sequences used are listed in Table S1. T24 cells were infected with lentivirus-packaged shRNAs, followed by selection with 2 μg/ml puromycin for one week. The depletion of genes was verified by both quantitative reverse-transcriptase-PCR (qRT-PCR) and western blotting. All RNA interference (RNAi) reagents were obtained from the National RNAi Core Facility, Academia Sinica, Taiwan.

Western blotting

Cells were harvested and lysed in lysis buffer (50 mM Tris, pH7.5, 150 mM NaCl, 1 mM EDTA, 0.1% Triton X-100). Protease inhibitor cocktail (MD Biol) was added to the lysis buffer immediately before lysis. Following gel electrophoresis and transfer onto PVDF membranes, the PVDF membranes were incubated for 1 hour or overnight in blocking buffer (5% milk in TBST [20 mM Tris, pH 7.6, 150 mM NaCl, 0.1% Tween 20]). Subsequently, the membranes were incubated with primary antibodies diluted in TBST for 3 hours at room temperature or overnight at 4°C, followed by incubation with the appropriate horseradish peroxidase (HRP)-conjugated secondary antibodies at room temperature for 1 hour. The primary antibodies used in this study are listed in Table S2. Images were detected using a chemiluminescence imaging system (GeneGnome 5 Bio Image, Syngene).

Plasmid constructs

The pLNCX-PARP1-GFP was generated by cloning the PARP1-GFP gene derived from the pEGFP-N1-PARP1 plasmid into pLNCX vector using In-Fusion HD Cloning Kit (Takara Bio). In order to eliminate the possibility that the exogenous PARP1 was

cleaved by sgRNA-guided Cas9 endonucleases, the sgRNA resistant PARP1-GFP, pLNCX-PARP1-GFP-Res, was generated using In-Fusion HD Cloning Kit (Takara Bio) with two primers, 5' TCG CGT GGA ATA TGC CAA GAG CGG GCG CGC C 3' and 5' GCA TAT TCC ACG CGA TAG AGC TTA TCC GAA GAC TCC G 3' (sgRNA resistant primers). The pLNCX-PARP1-K893I-GFP-Res plasmid, carrying the sgRNA resistant PARP1-K893I mutant was also generated using In-Fusion HD Cloning Kit (Takara Bio) with two primers 5' GTT TGG TAT AGG GAT CTA TTT CGC TGA CAT GG 3' and 5' ATC CCT ATA CCA AAC ATG TAG CCT GTC ACG G 3' (K893I primers). All of these constructs were verified by DNA sequencing.
FLAG-SHPRH (1-605), FLAG-SHPRH (606-1090), and FLAG-SHPRH (1090-1683) were generated using In-Fusion HD Cloning Kit (Takara Bio). FLAG-SHPRH (1-605) was generated with two primers, 5' CTG TCC AGC TAC TAG CGC TCG AGG TGA CTA CA 3' and 5' TGT AGT CAC CTC GAG CGC TAG TAG CTG GAC AG 3', FLAG-SHPRH (606-1090) was generated with two primers, 5' GAC AAG CTT GCG GCC TTG CGT GAT GGC CGA CTT G 3' and 5' ATG CCA CCC GGG ATC TCA CGA TGC GGC CGC TTC 3'. FLAG-SHPRH (1090-1683) were generated with two primers, 5' GAC AAG CTT GCG GCC TTG CGT GAT GGC CGA CTT G 3' and 5' ATG CCA CCC GGG ATC TCA CGA TGC GGC CGC TTC3'. All of these constructs were verified by DNA sequencing.
GFP-PARP1 (1-374) and GFP-PARP1 (529-1014) were generated using In-Fusion HD Cloning Kit (Takara Bio). GFP-PARP1 (1-374) was generated with two primers, 5' CGC CTC CGC CCT CCA CAG CCC GGG ATC CAC CGG T 3' and 5' GAC CGG TGG ATC CCG GGC TGT GGA GGG CGG AGG CG 3'. GFP-PARP1 (529-1014) was generated with two primers, 5' TCG AGA TGG GAG GAG CAG CTG TGG ATC C 3' and 5' CTC CTC CCA TCT CGA GAT CTG AGT CCG G 3'.
GFP-PARP1 (356-532) was generated by cloning the PCR fragment containing

PARP1 (356-532) into the *Xho*I/*Sac*II sites of pEGFP-N1 vector. All of these constructs were verified by DNA sequencing.

Generation of PARP1-GFP and PARP1-K893I-GFP expressing cell lines

The pLNCX-PARP1-GFP-Res and pLNCX-PARP1-K893I-Res plasmids carrying the sgRNA resistant wild-type PARP1 and PARP1-K893I mutant GFP fusion genes, respectively, were packaged into retrovirus particles using the Retro-X^{TM} Universal Packaging System (Takara Bio) in GP2-293 cell line. Retrovirus-containing medium was harvested at 48 and 72 hours after transfection and filtered through a 0.22 μm PES membrane syringe filter to eliminate packaging cells. PARP1-KO T24 cells were infected with the retrovirus with the presence of Polybrene (8 μg/mL, Sigma). 1 mg/mL hygromycin was added after 48 hours infection to select infected cells. The expression of PARP1 and PARP1-K893I mutant were verified by western blotting.

Sister chromatid exchange

T24 cells were seeded at a density of 1 x 10^6 cells in a 100-mm culture dish and incubated with 9 μg/mL 5-bromodeoxyuridine (BrdU; Sigma-Aldrich) for 42 hours. Then, the cells were treated with 0.1 μg/ml colcemid (ThermoFischer Scientific, 15212012) for 1 hour to harvest metaphase chromosomes. Subsequently, the cells were trypsinized and washed once with PBS before incubation in 75 mM KCl at 37 °C for 10 minutes, followed by the addition of a fixing solution (methanol/acetic acid, 3:1). The fixed cells were dropped onto slides to spread the chromosomes. The slides were incubated with 75 μg/ml Hoechst 33258 (ThermoFisher Scientific, H1398) for 10 minutes at room temperature, followed by crosslinking with 1200 J/m2 of UV irradiation in saline-sodium citrate (SSC). Finally, the slides were stained in 4% Giemsa in Gurr's buffer for 4 minutes, rinsed with deionized water twice, and dried at

room temperature. Images of metaphase chromosomes were acquired by a Nikon Eclipse 80i fitted with Nikon Plan Apo λ 60x/1.40 Oil OFN25 DIC N2 objective and NIS Elements D4.30.02. For each cell line, 50 metaphase cells were analyzed to determine the number of SCE events.

EdU click reaction

Cells were seeded in medium onto 8-well plastic chambers slides (Millipore) at a concentration of 30,000 cells/well and grown overnight in a humidified incubator containing 5% CO_2 at 37 °C. We labeled cells in culture with 10 μM EdU for 15 minutes followed by damage treatment before fixation (we treated cells with 50 uM HU or 0.01% MMS for 1 hour). The cells were fixed in 3.5% paraformaldehyde in PBS for 30 minutes, washed three times with PBS, and subjected to the click reaction using 10 μM Cy5-azide, 10 mM sodium ascorbate, and 2 mM $CuSO_4$ in PBS for 30 minutes. Cells were rinsed in PBS twice and incubated with DAPI diluted in PBS for 15 minutes at room temperature. Images were acquired using a Zeiss LSM 780 confocal microscope, and Cy5 intensity was quantified with ZEN 3.3 (blue edition) in at least 100 cells per cell line.

The PLA assay

The PLA assay was performed according to the manufacturer's protocol (Duolink, Sigma-Aldrich). The primary antibodies used in this study are listed in Table S2. The samples were mounted with Duolink In Situ Mounting Medium with DAPI for 15 minutes at room temperature and analyzed using a Nikon eclipse 80i microscope equipped with a Plan Fluor 40x/0.75 DIC M/N2 objective. The resulting images were then measured using NIS Elements D4.20.00 software (Nikon).

Co-immunoprecipitation (CoIP) assay

6 x 10^6 cells were treated with either mock or 0.01% MMS for 1 hour. Cells were harvested and lysed in Tween lysis buffer (10% Glycerol, 100 mM KCl, 5mM $MgCl_2$, 20 mM Tris-HCl pH8.0, 0.2 mM EDTA, 0.1% Tween20, and protease inhibitor cocktail [MD Biol]). Cell lysates were then sonicated to shear chromosomal DNA. Following centrifugation, the supernatant containing protein complexes was transferred into a clean tube. To rule out the possibility that the interaction is mediated through DNA, the supernatant was treated with either 100 μg/mL DNase I at 37°C for 20 minutes or 50 μg/mL EtBr on ice for 30 minutes. The supernatant was precleaned using protein G-sepharose and then subjected to coimmunorprcipitation with a specific antibody at 4°C for 2 hours and followed by protein G-sepharose pulldown at 4°C for 1 hour. After being washed with Tween lysis buffer for 3 times, the immunoprecipitates were mixed with 2x Laemmli sample buffer and boiled for 5 minutes. Finally, the samples were resolved by a 8% SDS-PAGE, followed by western blotting analysis.
