# Supplementary figures and images for: PARP1 recruits DNA translocases to restrain DNA replication and facilitate DNA repair
Source: PLoS Genet. 2022 Dec 13;18(12):e1010545. doi: 10.1371/journal.pgen.1010545 (PMC9794062; doi:10.1371/journal.pgen.1010545)

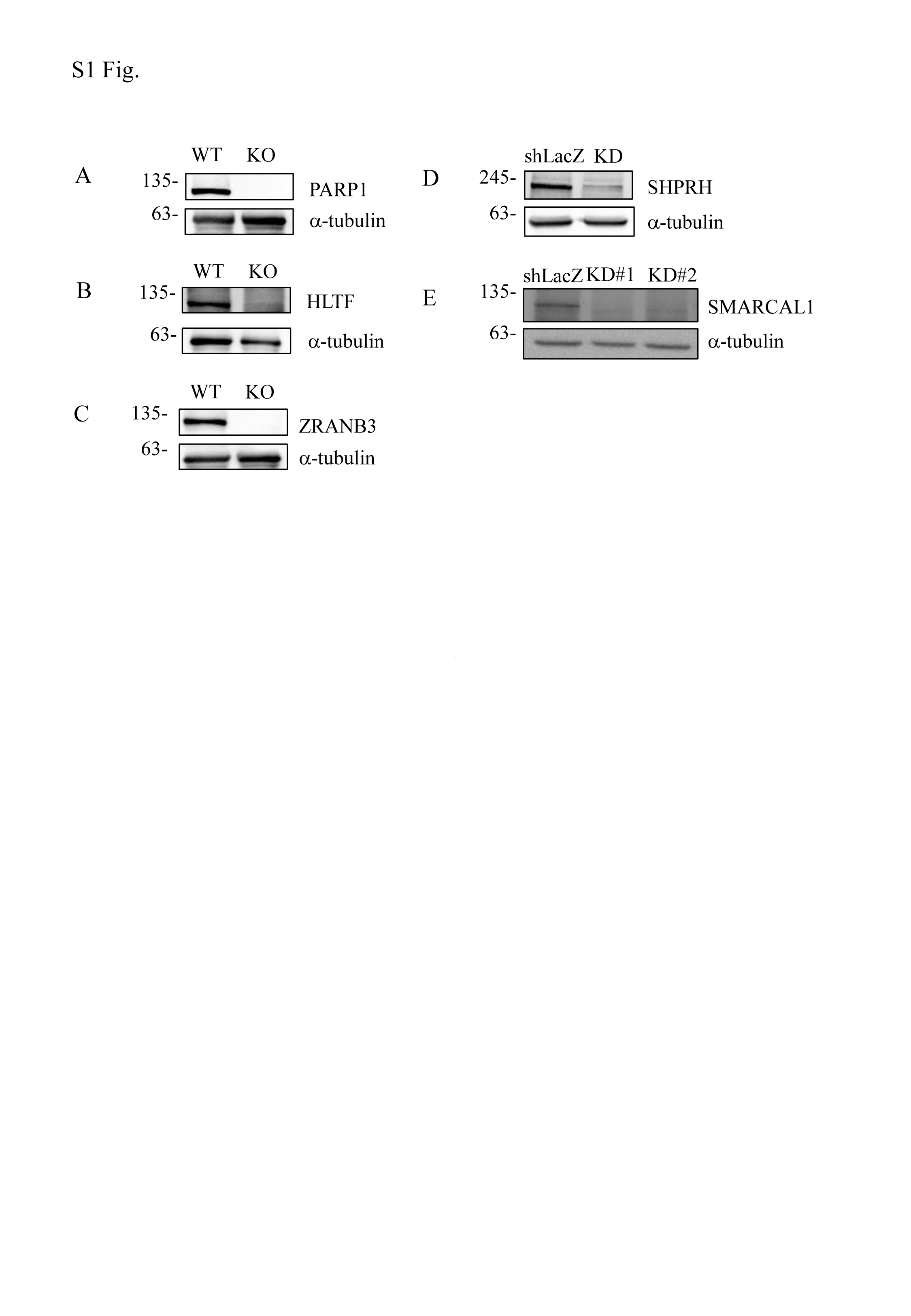

Supplement: S1 Fig — (A)-(E) The knockdown (KD) or knockout (KO) of each gene was verified by western blot analysis using specific antibodies as indicated. Two shRNAs, KD#1 and KD#2, against SMARCAL1 were used as indicated. (TIF) [file pgen.1010545.s002.tif]

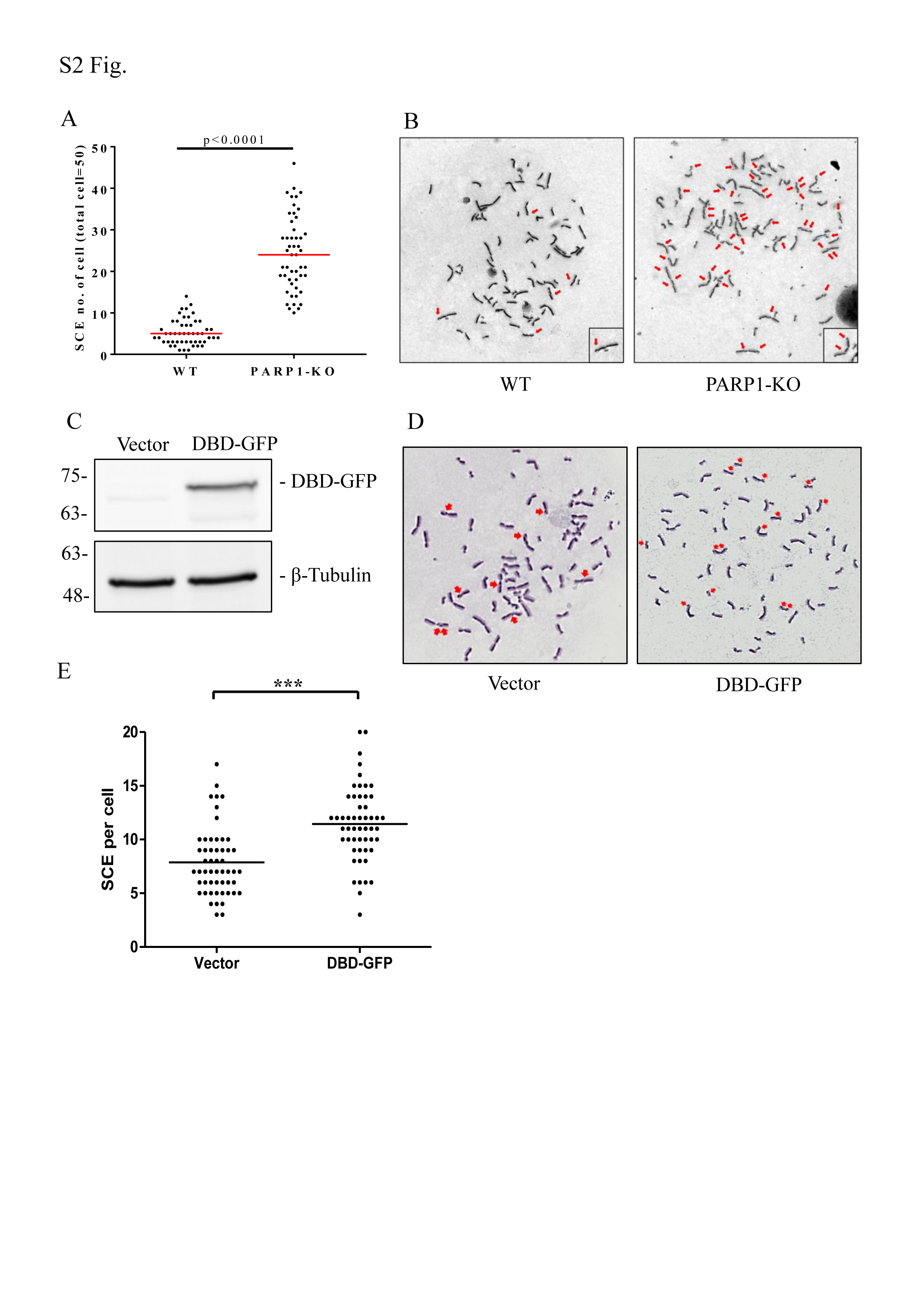

Supplement: S2 Fig — (A) SCE analysis of control T24 cells and PARP1-KO T24 cells. SCE was scored in 50 metaphase cells in each cell line. (B) Representative images of SCE derived from each cell line. (C) Overexpression of DNA binding domain of PARP1 increases SCE frequency. HONE6 cells were transfected with an empty vector pEGFP or pEGFP-DBD. The expression of DBD-GFP fusion protein was determined using western blotting with an anti-GFP antibody. (D) Representative images of SCE for each cell line. (E) Quantification of SCE was scored from at least 50 metaphase cells per cell line. (Raw SCE data in S8 Data). (TIF) [file pgen.1010545.s003.tif]

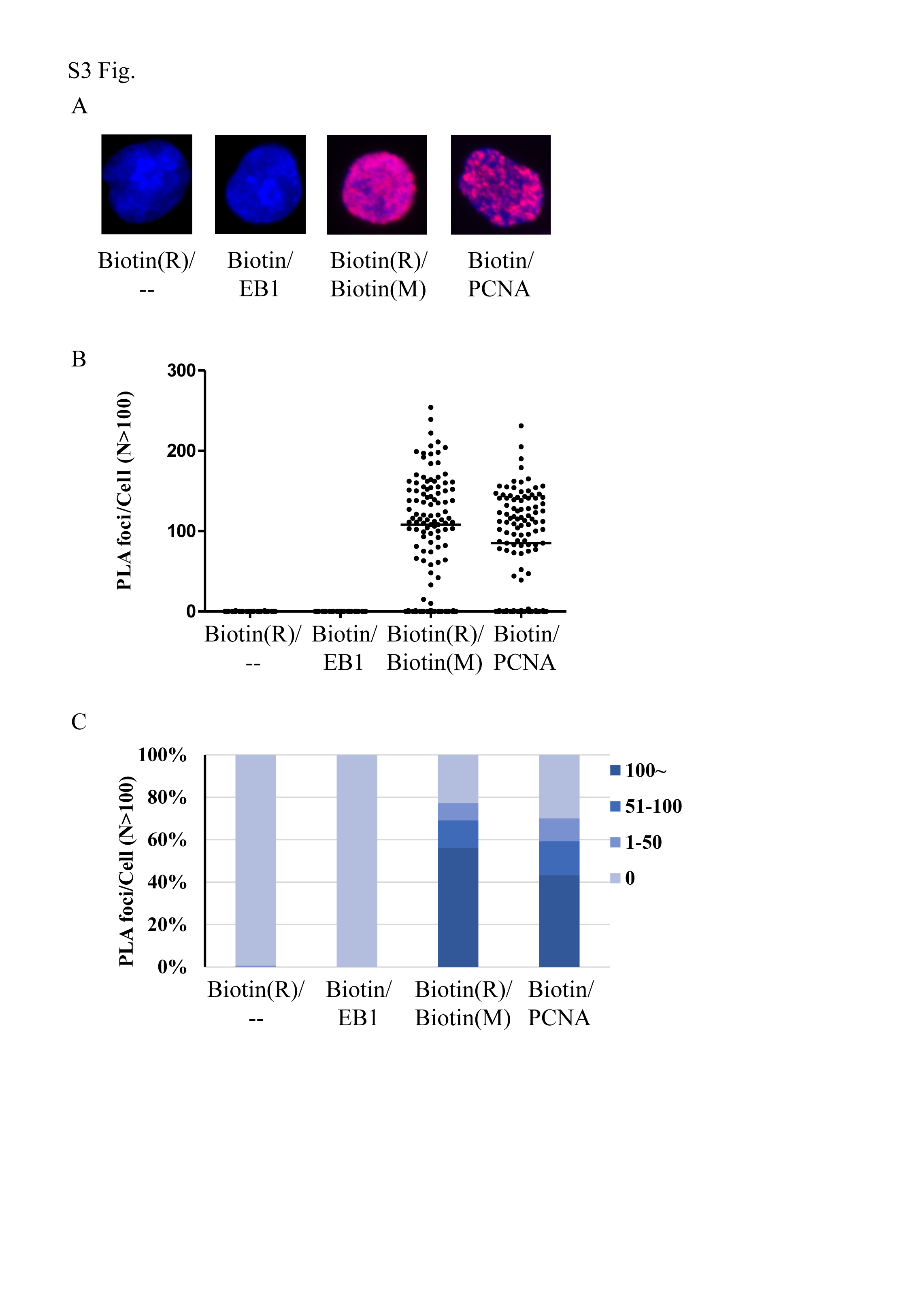

Supplement: S3 Fig — (A) Representative images of control PLA and PCNA SIRF assays. PLA foci are shown in red, and DAPI staining is shown in blue. (B) Distribution of PLA foci from each cell derived from (A). At least 100 cells from each condition were measured. (C) The numbers of PLA foci from each cell were classified into four groups: 0, 1–50, 51–100, and >100 foci. The distributions of each group are indicated in the plot. (Raw SIRF data in S9 Data). (TIF) [file pgen.1010545.s004.tif]

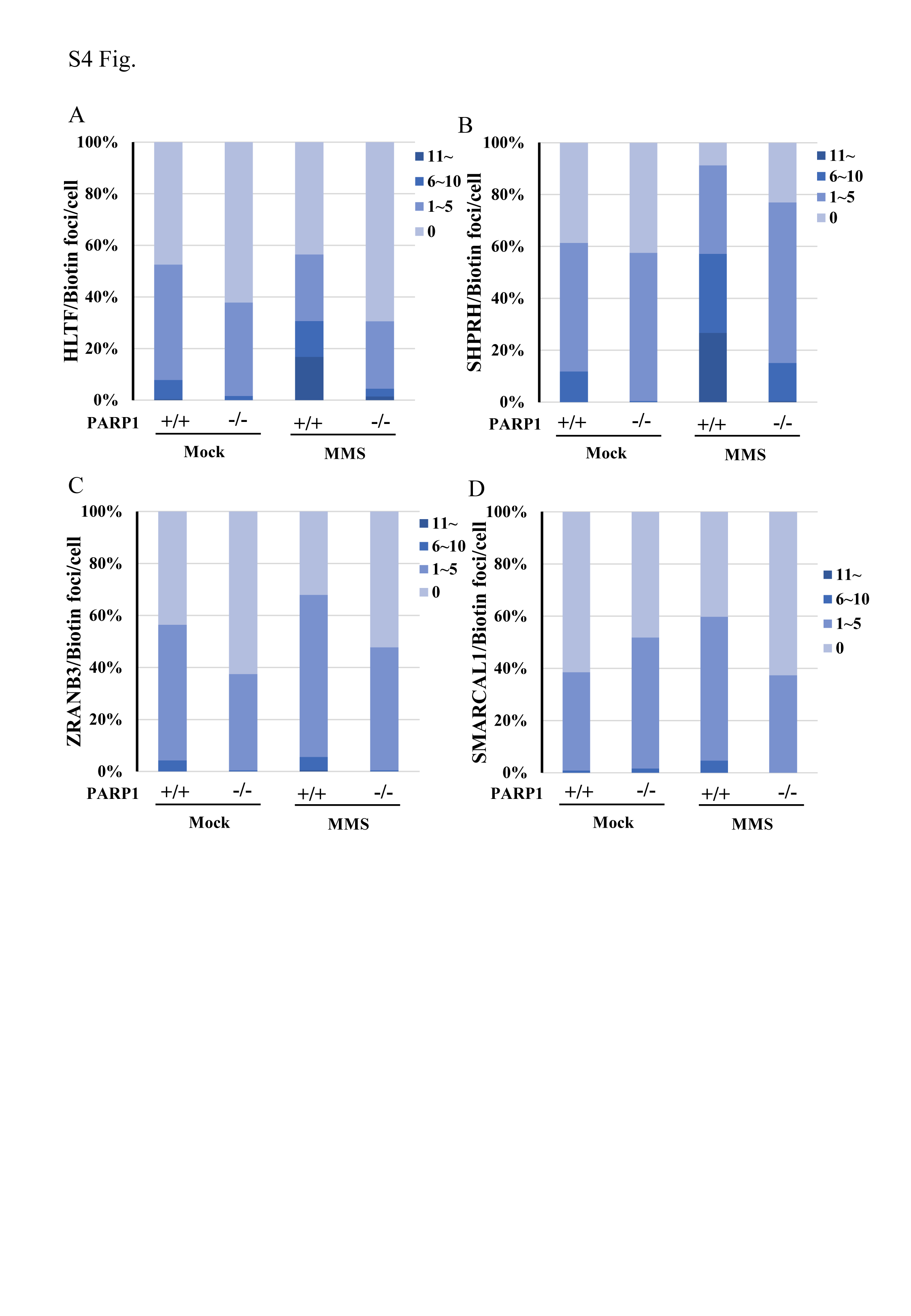

Supplement: S4 Fig — (A)-(D) The numbers of PLA foci (Fig 1A–1D) were classified into four groups: 0, 1–5, 6–10, and >11 foci, and the distributions of each group are shown in the plots. (Raw SIRF data in S1 Data). (TIF) [file pgen.1010545.s005.tif]

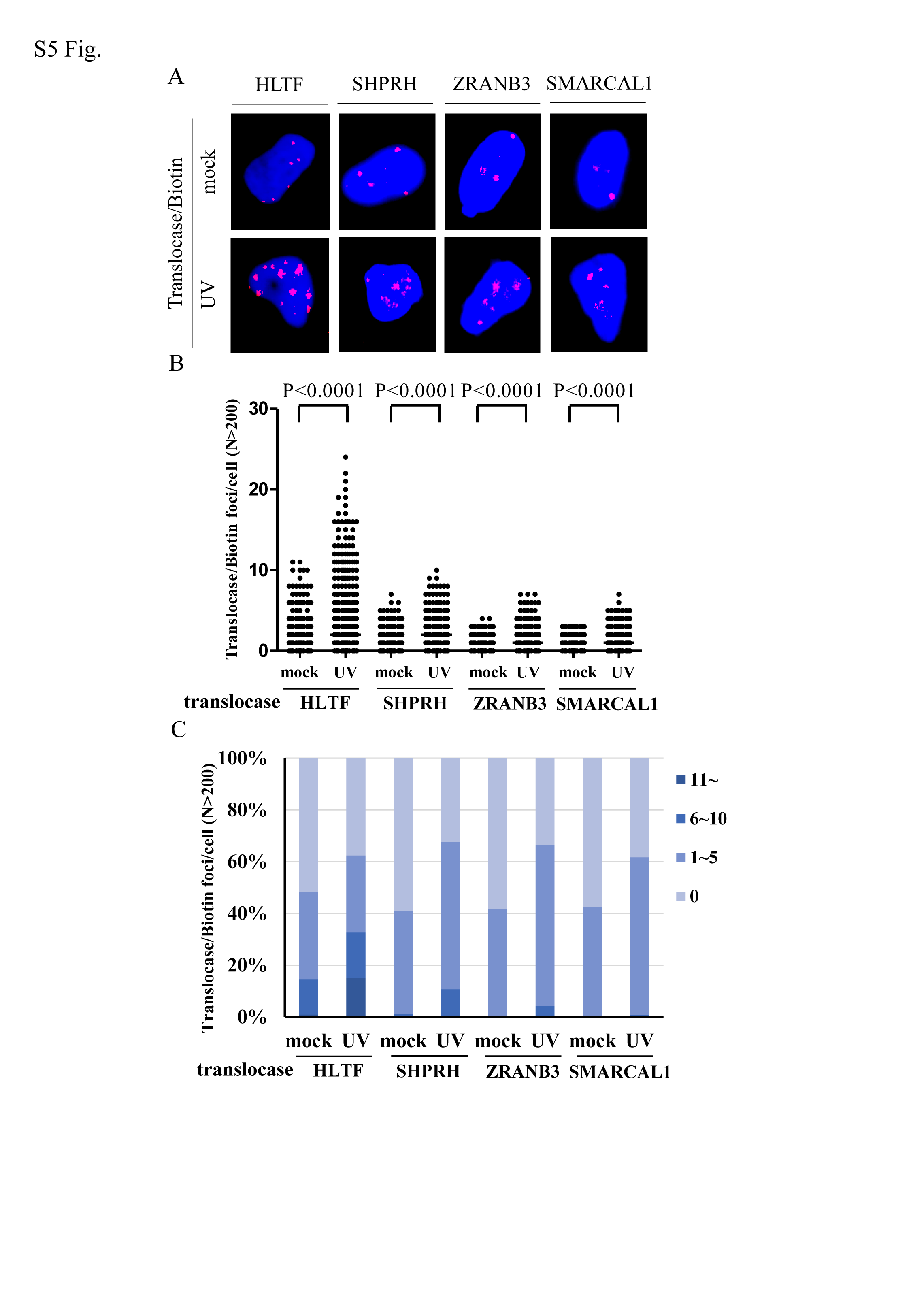

Supplement: S5 Fig — (A) Representative images of PLA foci of each DNA translocase in mock or UV-treated T24 cells. Cells were treated with 60 J/cm2 of UV irradiation. The association of each DNA translocase with replication forks was determined by the SIRF assay. (B) Distribution of PLA foci derived from a, respectively. At least 200 cells from each condition were measured. (C) The number of PLA foci was classified into four groups: 0, 1–5, 6–10, and >11 foci, and the distributions of each group were shown in the plot. (Raw SIRF data in S10 Data). (TIF) [file pgen.1010545.s006.tif]

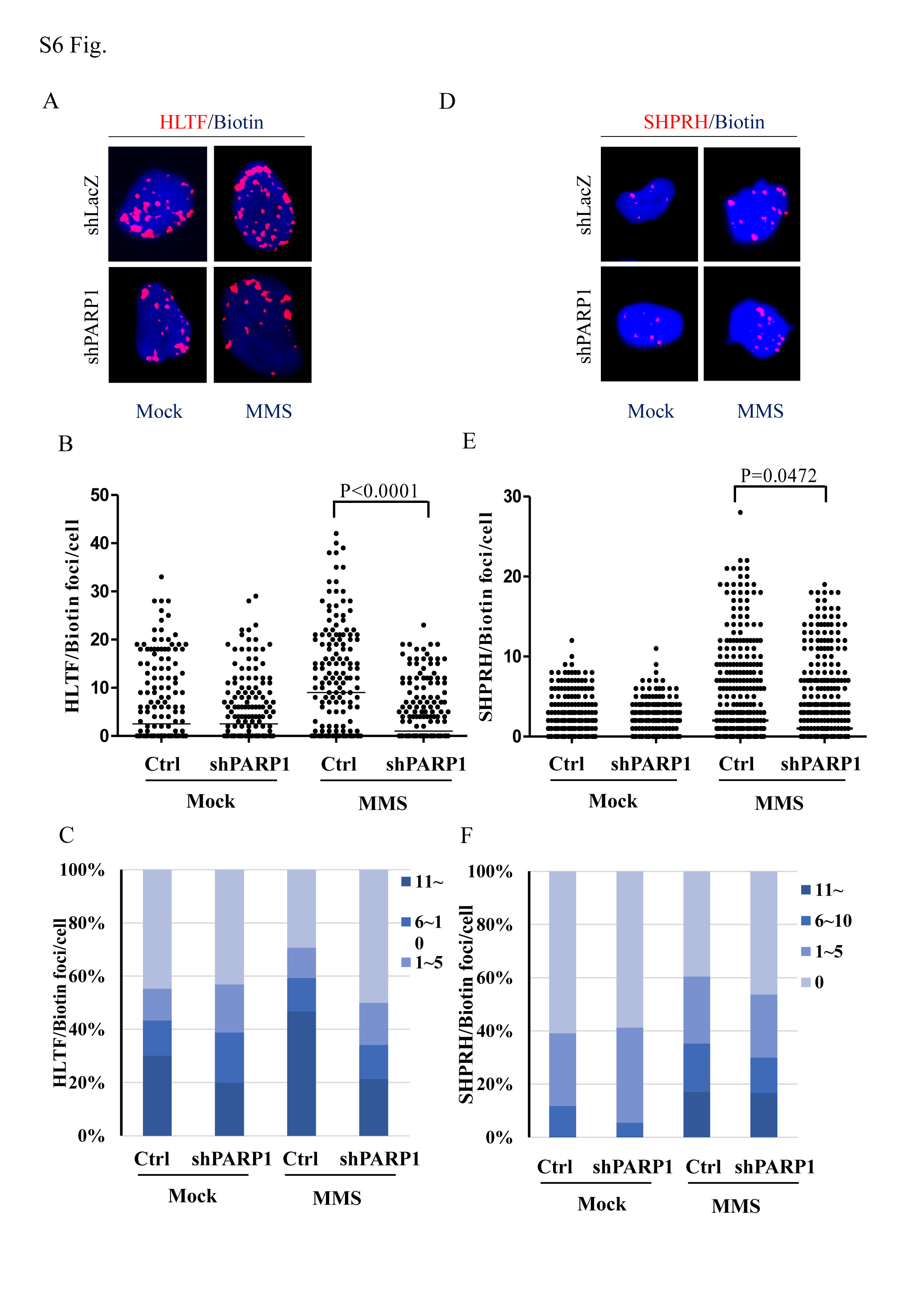

Supplement: S6 Fig — (A)(D) Representative images of HLTF and SHPRH PLA foci in wild-type and PARP1-knockdown T24 cells. The expression of PARP1 was depleted using shRNA lentivirus. Cells were treated with 0.01% MMS for 1 hour. The association of each protein with replication forks was determined by the SIRF assay. (B)(E) Distribution of HLTF and SHPRH PLA foci derived from (A)(D), respectively. At least 200 cells from each condition were measured. (C)(F) The numbers of PLA foci were classified into four groups: 0, 1–5, 6–10, and >11 foci, and the distributions of each group are shown in the plot. (Raw SIRF data in S11 Data). (TIF) [file pgen.1010545.s007.tif]

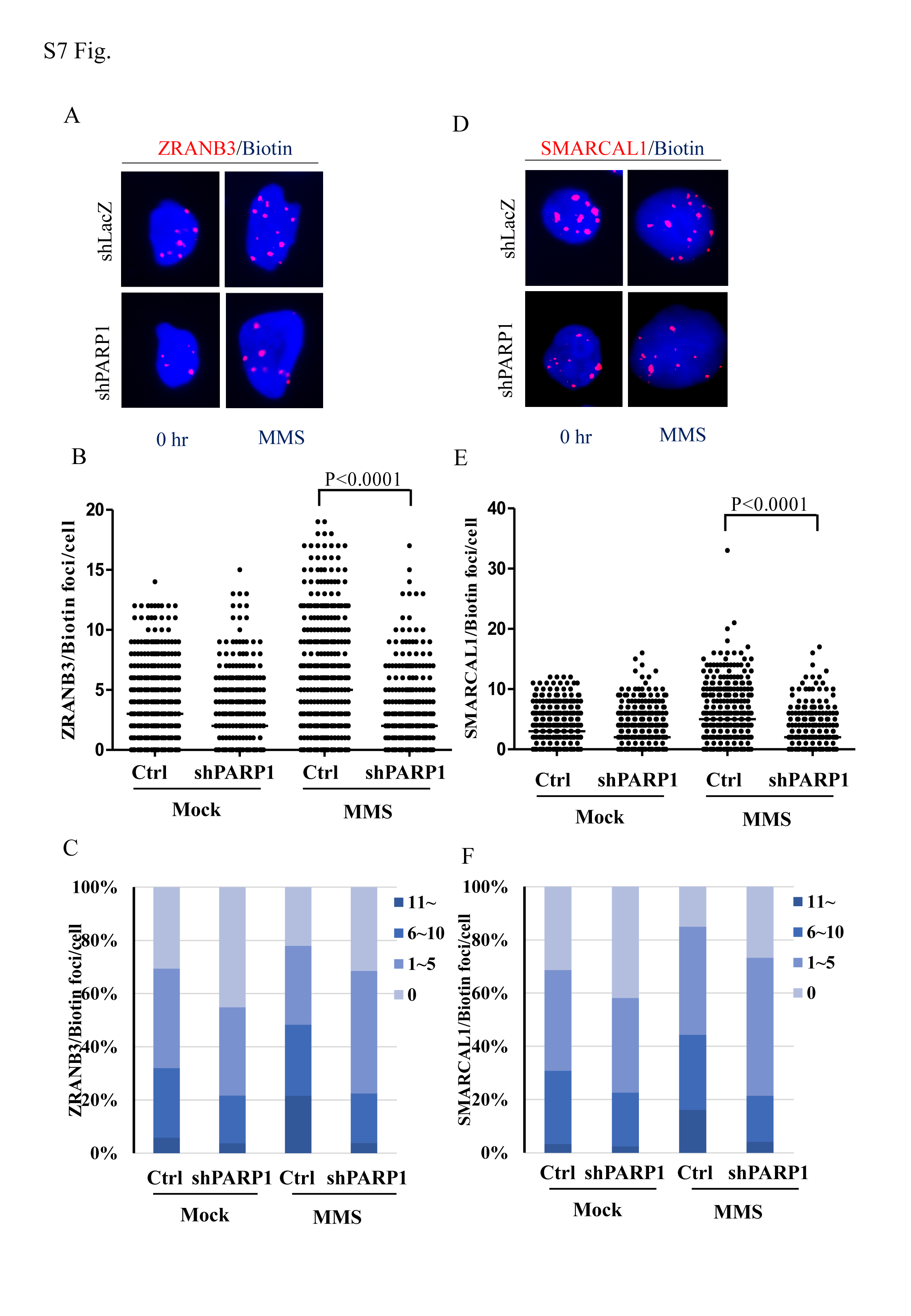

Supplement: S7 Fig — (A)(D) Representative images of ZRANB3 and SMARCAL1 PLA foci in wild-type and PARP1-knockdown T24 cells. The expression of PARP1 was depleted using shRNA lentivirus. Cells were treated with 0.01% MMS for 1 hour. The association of each protein with replication forks was determined by the SIRF assay. (B)(E) Distribution of ZRANB3 and SMARCAL1 PLA foci derived from (A)(D), respectively. At least 200 cells from each condition were measured. (C)(F) The numbers of PLA foci were classified into four groups: 0, 1–5, 6–10, and >11 foci, and the distributions of each group are shown in the plot. (Raw SIRF data in S11 Data). (TIF) [file pgen.1010545.s008.tif]

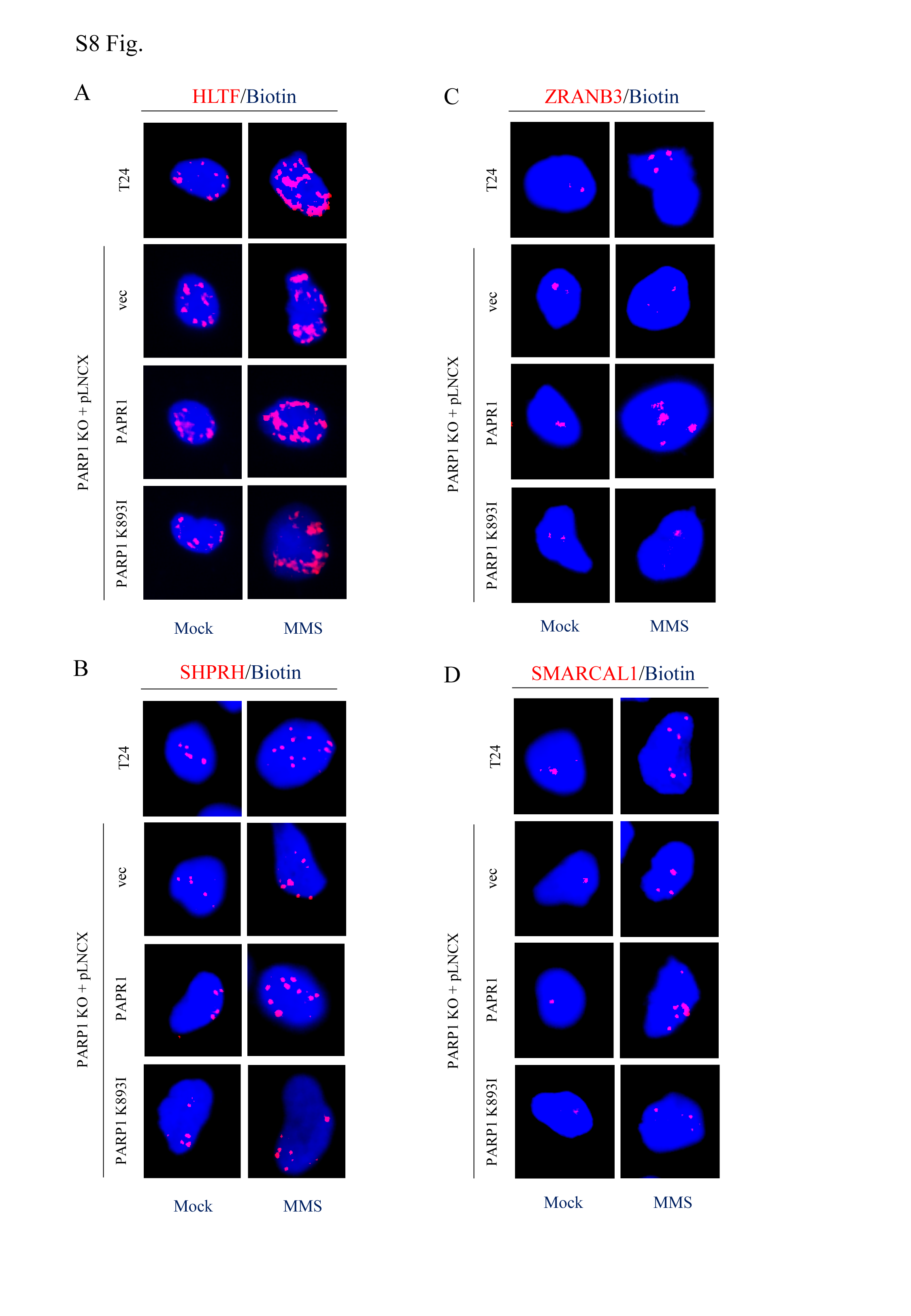

Supplement: S8 Fig — (A)-(D) Representative images of HLTF, SHPRH, ZRANB3, and SMARCAL1 PLA foci, respectively, in PARP1-KO, PAPR1-rescue, and PARP1-K893I expressing T24 cells. The pLNCX vectors carrying wild-type PARP1 or PARP1-K893I mutant, respectively, were packaged into retrovirus particles in GP2-293 cell line. PARP1-KO T24 cells were infected with these retroviruses to stably express wild-type PARP1 or PARP1-K893I mutant. Retrovirus carrying the empty vector (vec) was used as the control. Cells were treated with 0.01% MMS for 1 hour. The association of each protein with replication forks was determined by the SIRF assay. (Raw SIRF data in S2 Data). (TIF) [file pgen.1010545.s009.tif]

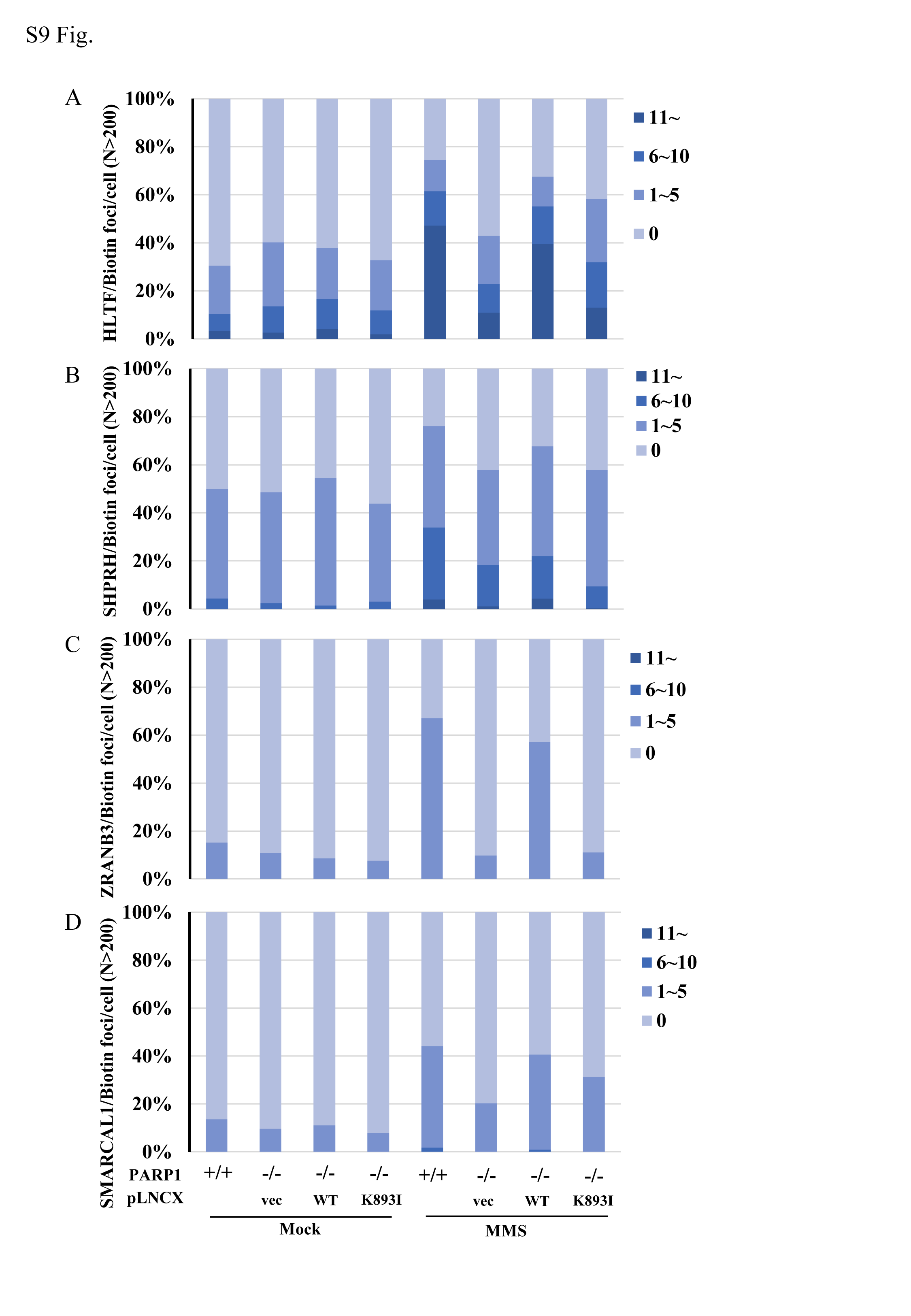

Supplement: S9 Fig — (A)-(D) The numbers of PLA foci were classified into four groups: 0, 1–5, 6–10, and >11 foci, and the distributions of each group are shown in the plot. (Raw SIRF data in S2 Data). (TIF) [file pgen.1010545.s010.tif]

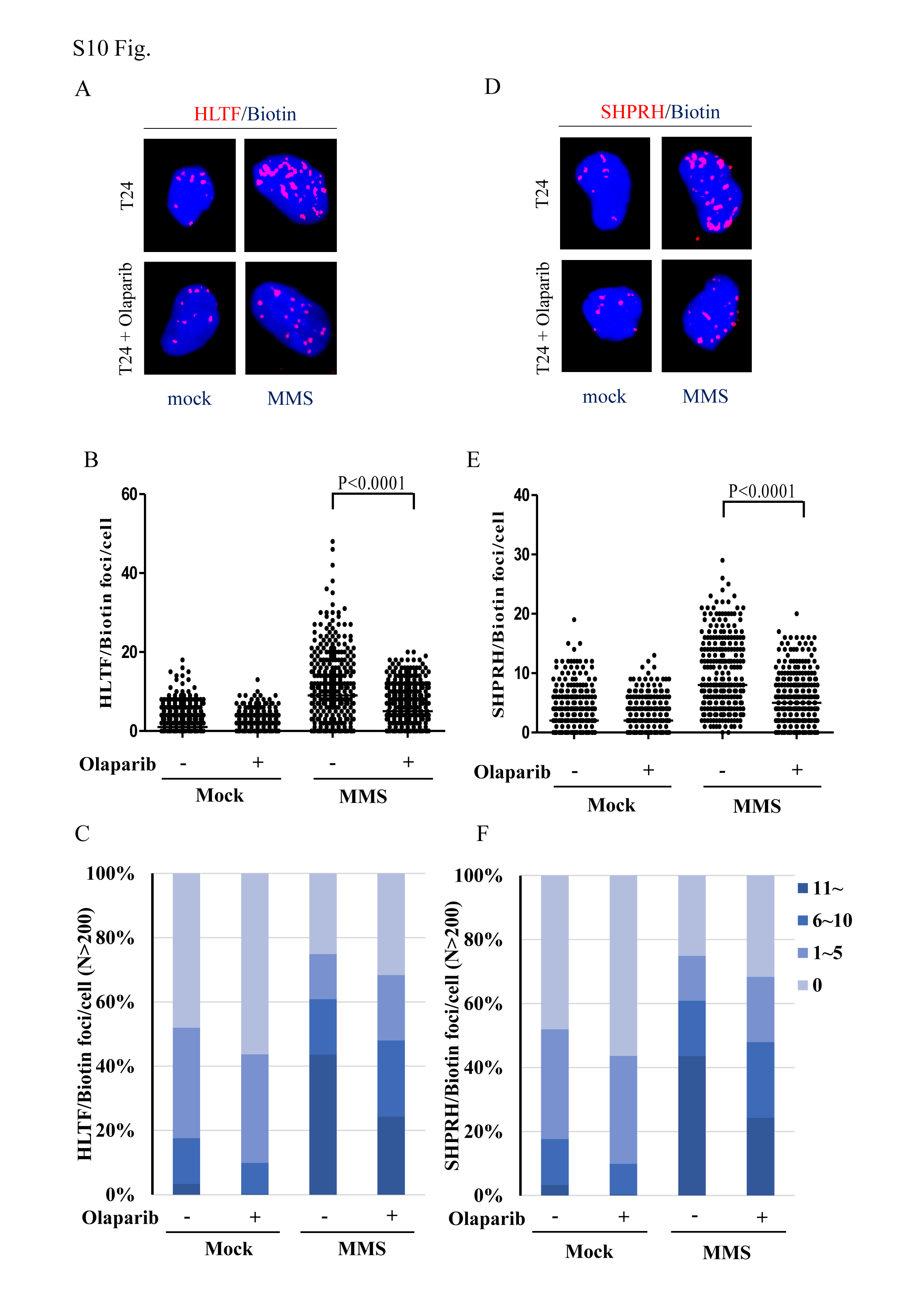

Supplement: S10 Fig — (A)(D) Representative images of HLTF and SHPRH PLA foci in mock or olaparib-treated T24 cells. Cells were treated with olaparib for 2 hours, followed by 0.01% MMS treatment for 1 hr. The association of each protein with replication forks was determined by the SIRF assay. (B)(E) Distributions of HLTF and SHPRH-PLA foci derived from a, d, respectively. At least 200 cells from each condition were measured. (C)(F) The numbers of PLA foci were classified into four groups: 0, 1–5, 6–10, and >11 foci, and the distributions of each group are shown in the plot. (Raw SIRF data in S12 Data). (TIF) [file pgen.1010545.s011.tif]

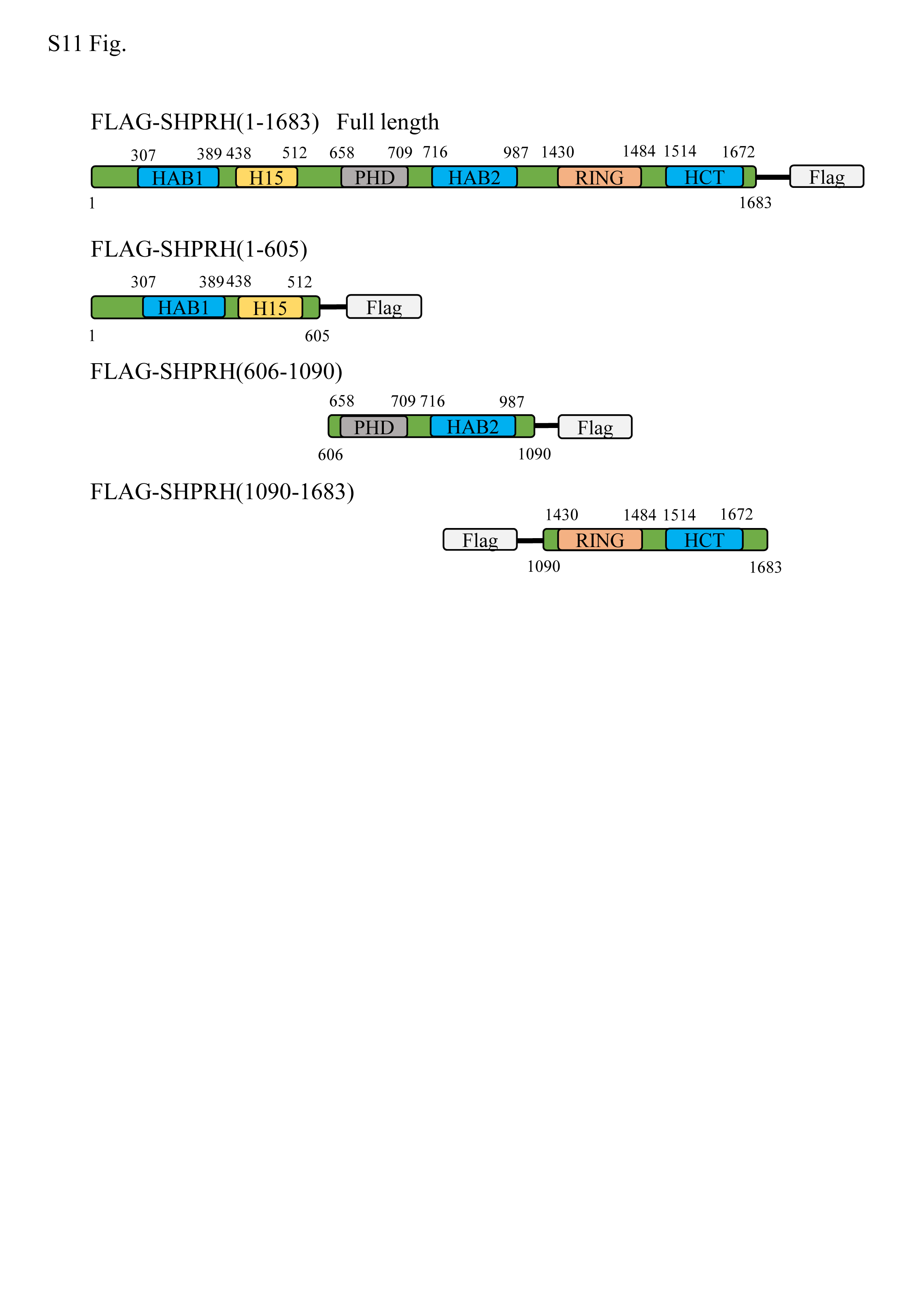

Supplement: S11 Fig — The helicase ATP binding domain first part (HAB1), H15, PHD, helicase ATP binding domain second part (HAB2), RING, and helicase C-terminal domain (HCT) are based on UniProt Knowledgebase (UniProtKB) analysis. (TIF) [file pgen.1010545.s012.tif]

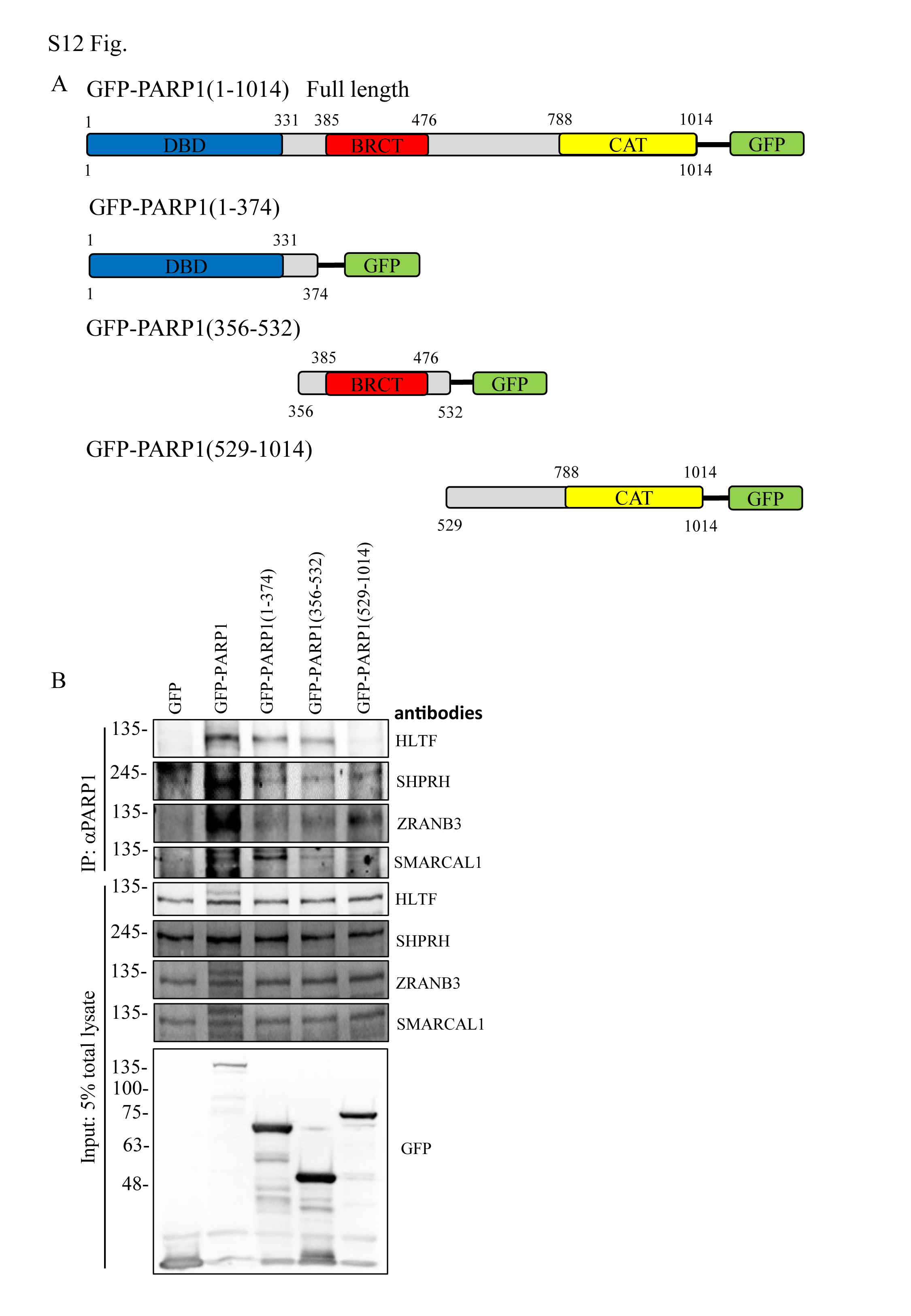

Supplement: S12 Fig — (A) The schematic representation of PARP1 constructs. The DNA binding domain (DBD), BRCT domain, and catalytic domain (CAT) are based on UniProt Knowledgebase (UniProtKB) analysis. (B) HEK293T cells were transfected with various GFP-PARP1 constructs. The GFP-PARP1 fusion proteins were immunoprecipitated with a GFP antibody followed by protein G-agarose pulldown. The immunoprecipitates were then subjected to immunoblotting analysis with specific antibodies as indicated. Input represents 5% of total cell lysates. (TIF) [file pgen.1010545.s013.tif]

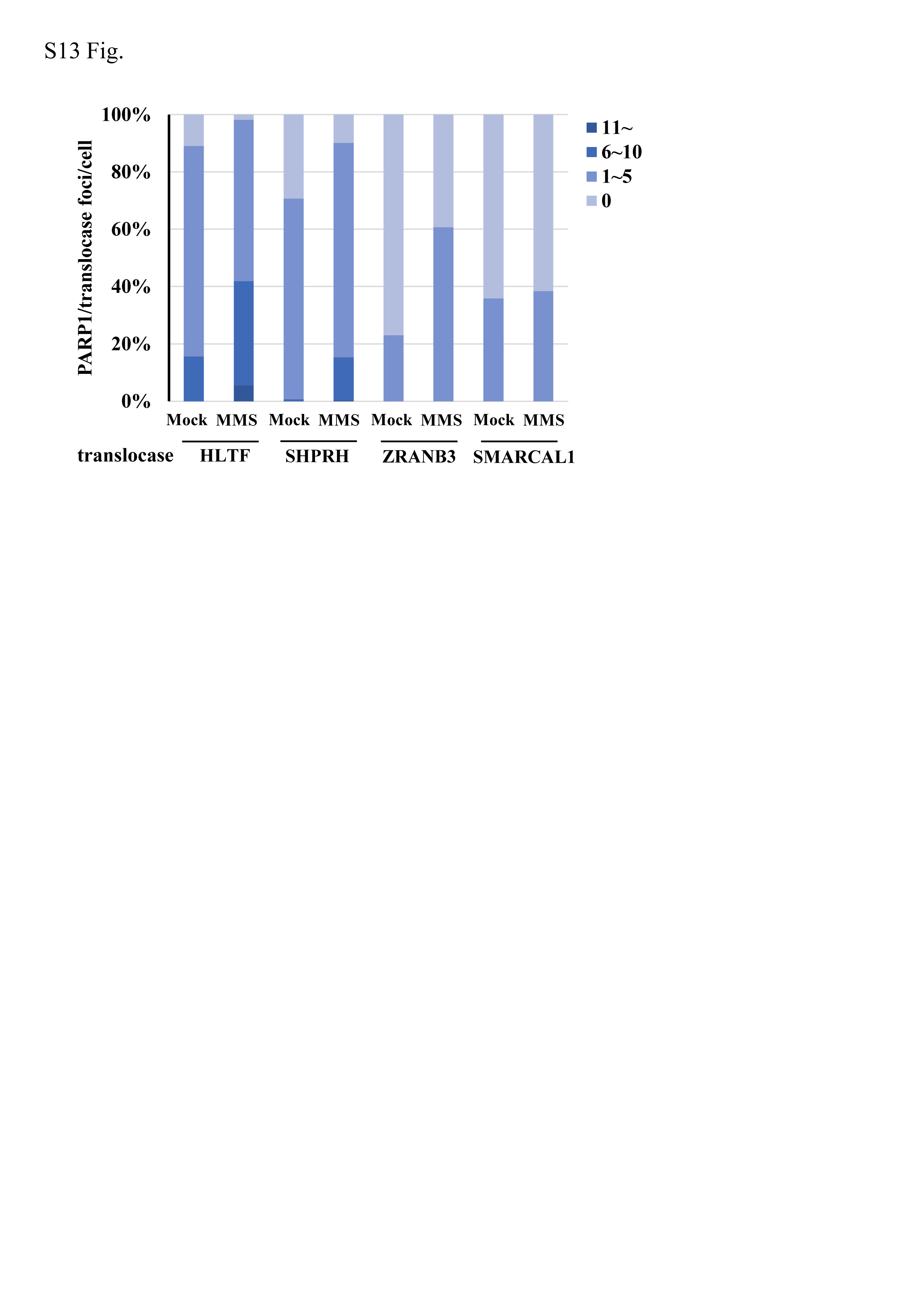

Supplement: S13 Fig — The number of PLA foci (Fig 3C) was classified into four groups: 0, 1–5, 6–10, and >11 foci, and the distributions of each group are shown in the plot. (Raw PLA data in S3 Data). (TIF) [file pgen.1010545.s014.tif]

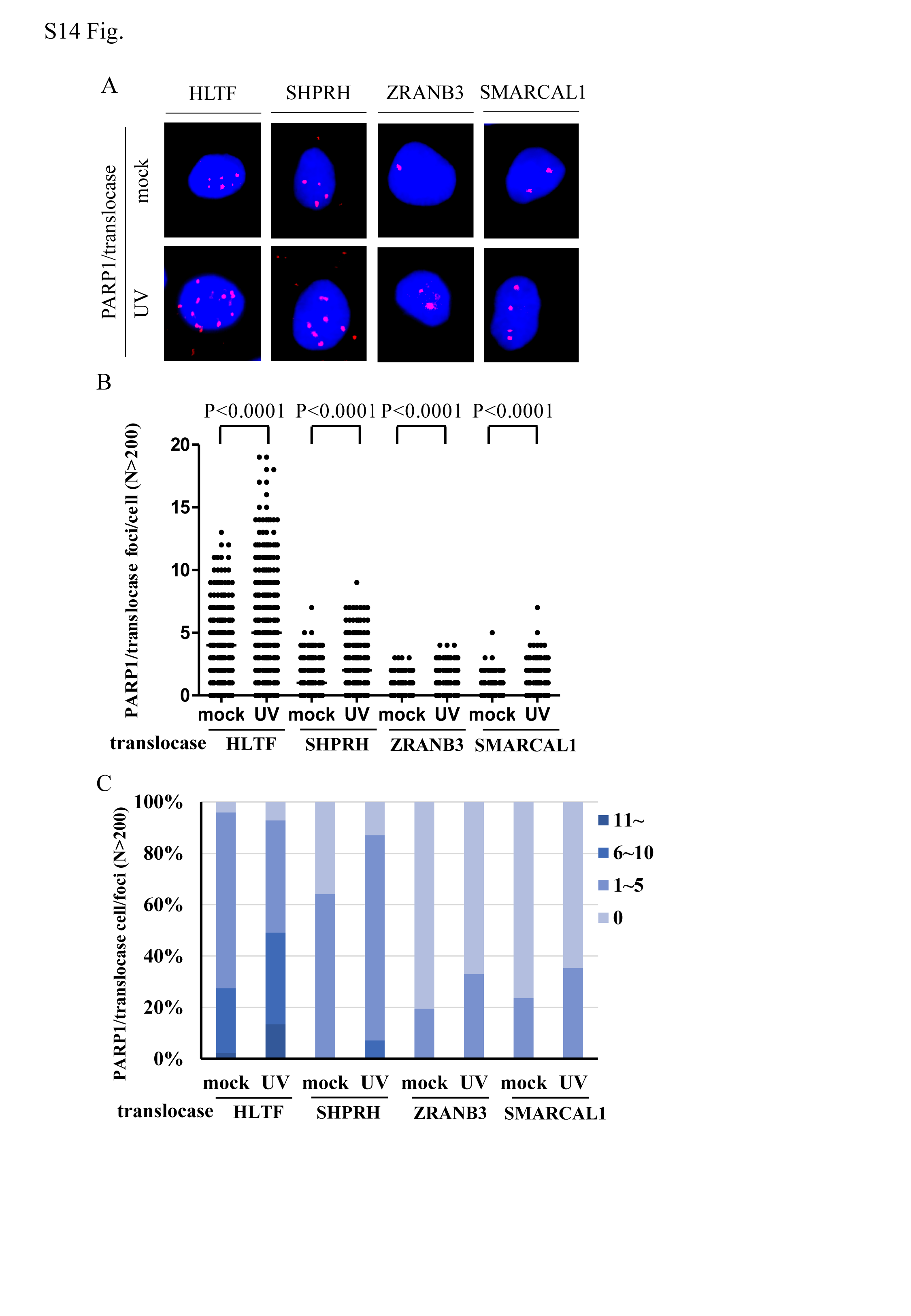

Supplement: S14 Fig — (A) Representative images of PARP1/translocases PLA foci in mock or UV-treated T24 cells. T24 cells were treated with 60 J/cm2 of UV irradiation. (B) Distributions of PARP1/HLTF, PARP1/SHPRH, PARP1/ZRANB3, and PARP1/SMARCAL1 PLA foci derived from a. At least 200 cells from each condition were measured. (C) The numbers of PLA foci were classified into four groups: 0, 1–5, 6–10, and >11 foci, and the distributions of each group are shown in the plot. (Raw PLA data in S13 Data). (TIF) [file pgen.1010545.s015.tif]

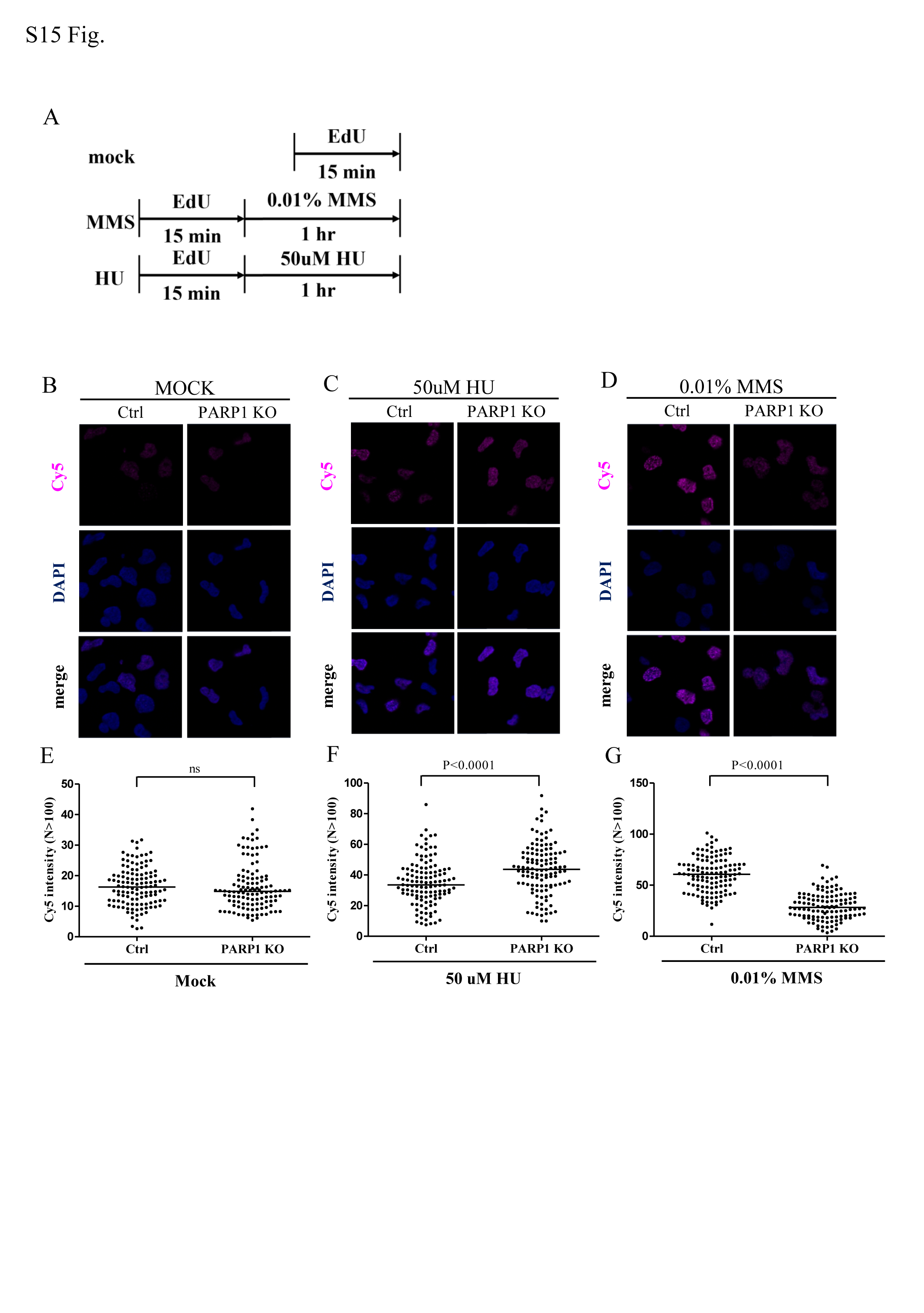

Supplement: S15 Fig — (A) The schematic representation of EdU click reaction assay. (B)(C)(D) Representative images of Cy5 fluorescent intensity derived from each cell line and treatment. Wild-type and PARP1-KO T24 cells were labeled with EdU for 15 min (mock), followed by treatment with 50 μM HU or 0.01% MMS for 1 hour before fixation. Cy5 (pink) was conjugated to EdU by the click reaction, and images were acquired using a Zeiss LSM 780 confocal microscope. (E)(F)(G) The intensity of Cy5 was quantified from at least 100 cells per condition. (Raw Cy5 data in S14 Data). (TIF) [file pgen.1010545.s016.tif]

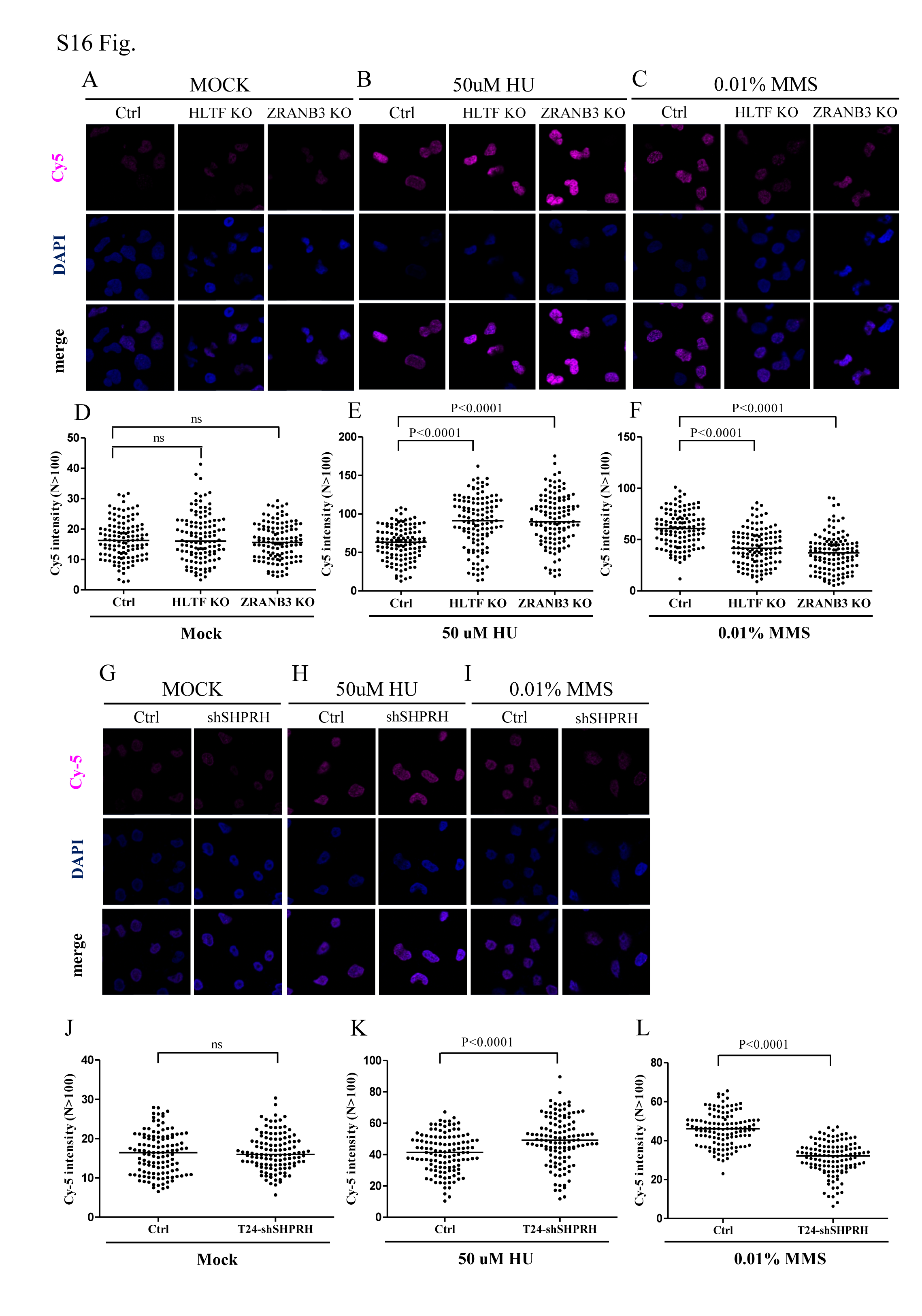

Supplement: S16 Fig — (A)(B)(C)(G)(H)(I) Representative images of Cy5 fluorescent intensity derived from each cell line and treatment. The wild type, HLTF-KO, ZRANB3-KO, and SHPRH-knockdown (shSHPRH) T24 cells were labeled with EdU for 15 min (mock), followed by treatment with 50 μM HU or 0.01% MMS for 1 hour before fixation. Cy5 (pink) was conjugated to EdU by the click reaction, and images were acquired using a Zeiss LSM 780 confocal microscope. (D)(E)(F)(J)(K)(L) The intensity of Cy5 was quantified from at least 100 cells per condition. (Raw Cy5 data in S14 Data). (TIF) [file pgen.1010545.s017.tif]

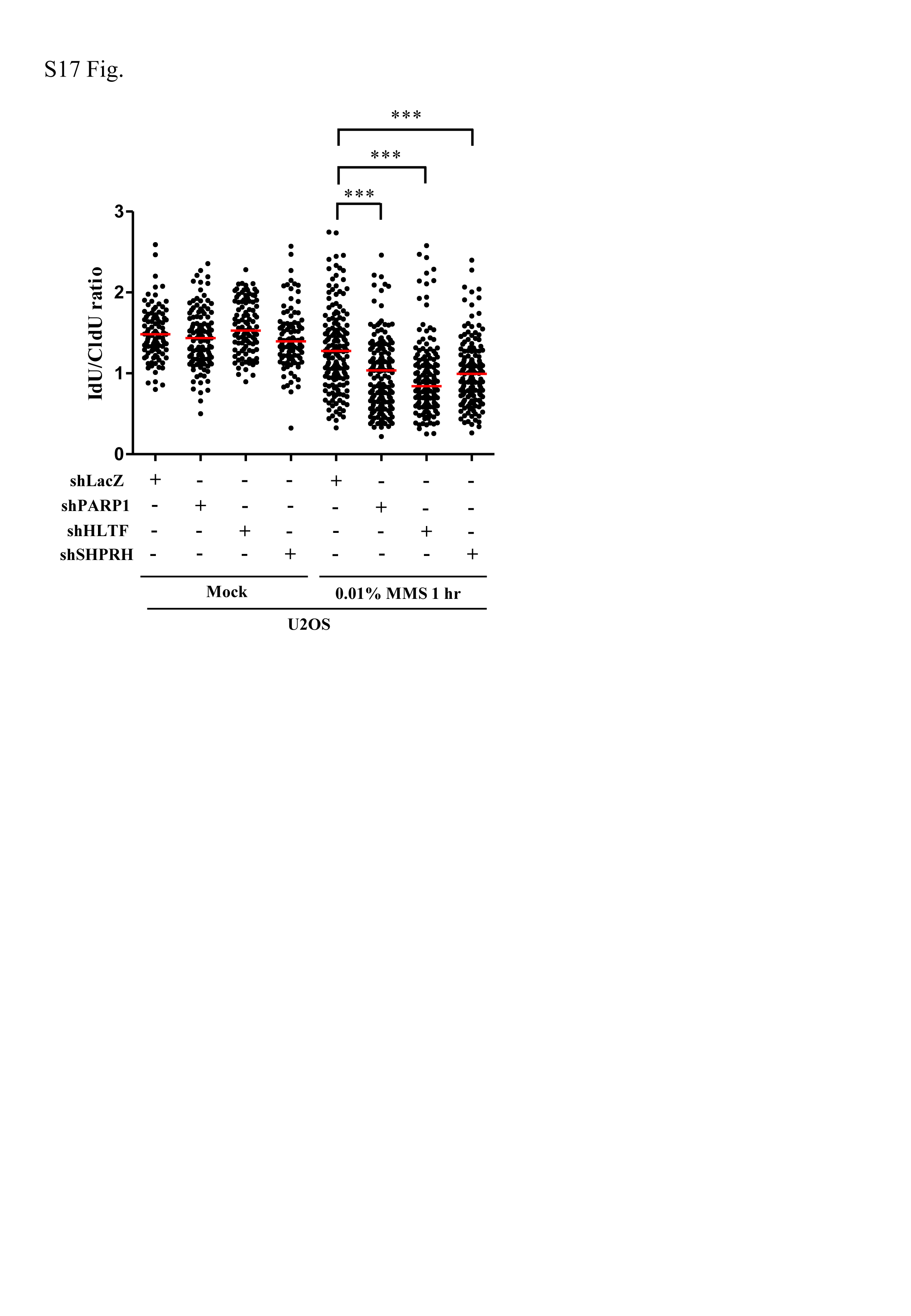

Supplement: S17 Fig — Quantitation of IdU/CldU track length ratios derived from each cell line. At least 100 DNA fibers derived from each cell line were measured. (Raw DNA fiber data in S15 Data). (TIF) [file pgen.1010545.s018.tif]

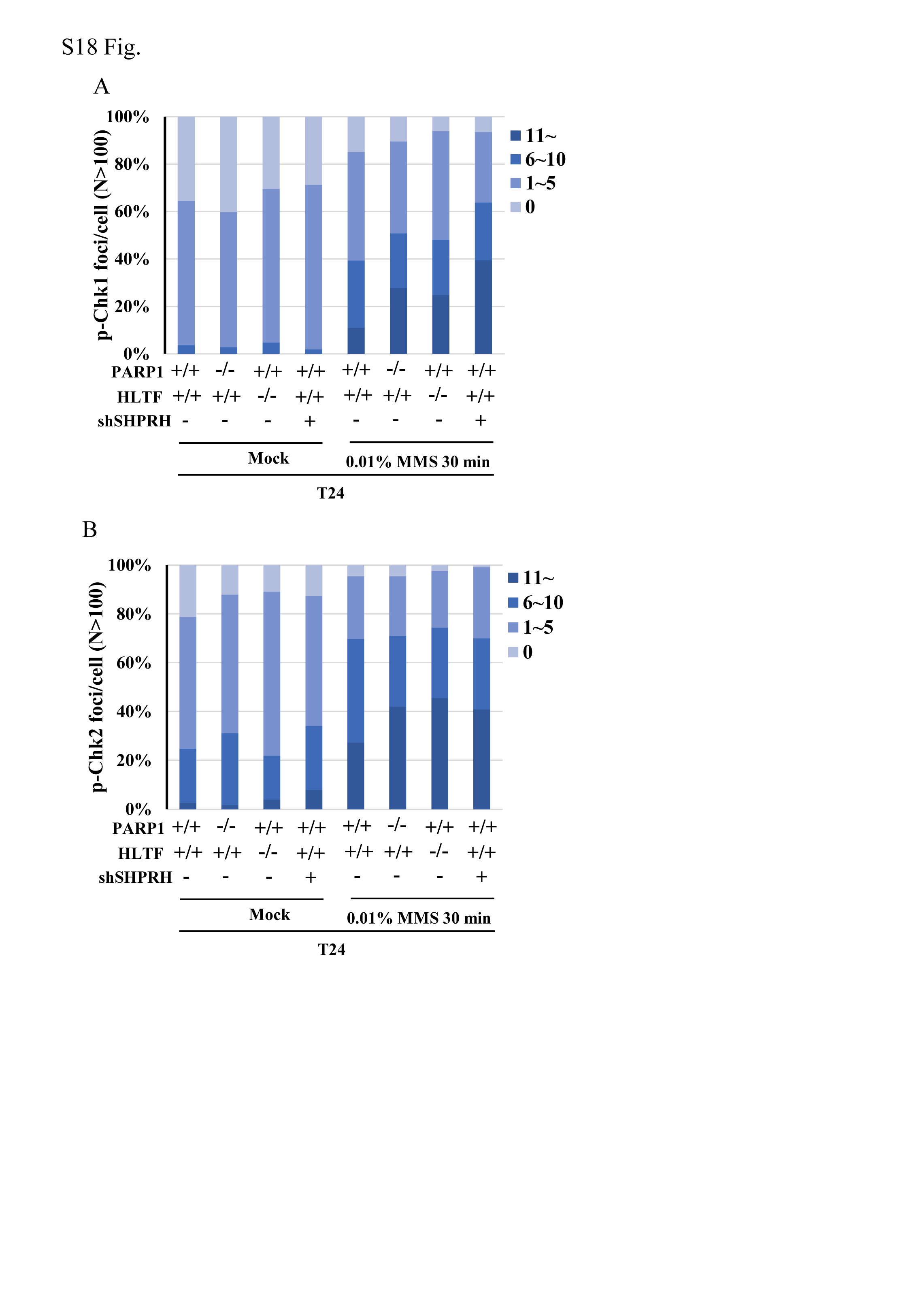

Supplement: S18 Fig — (A)(B) The numbers of phospho-CHK1 and phospho-CHK2 foci from each cell were classified into four groups: 0, 1–5, 6–10, >11 foci. The distributions of each group are indicated in the plot. (Raw pCHK1/pCHK2 data in S6 Data). (TIF) [file pgen.1010545.s019.tif]

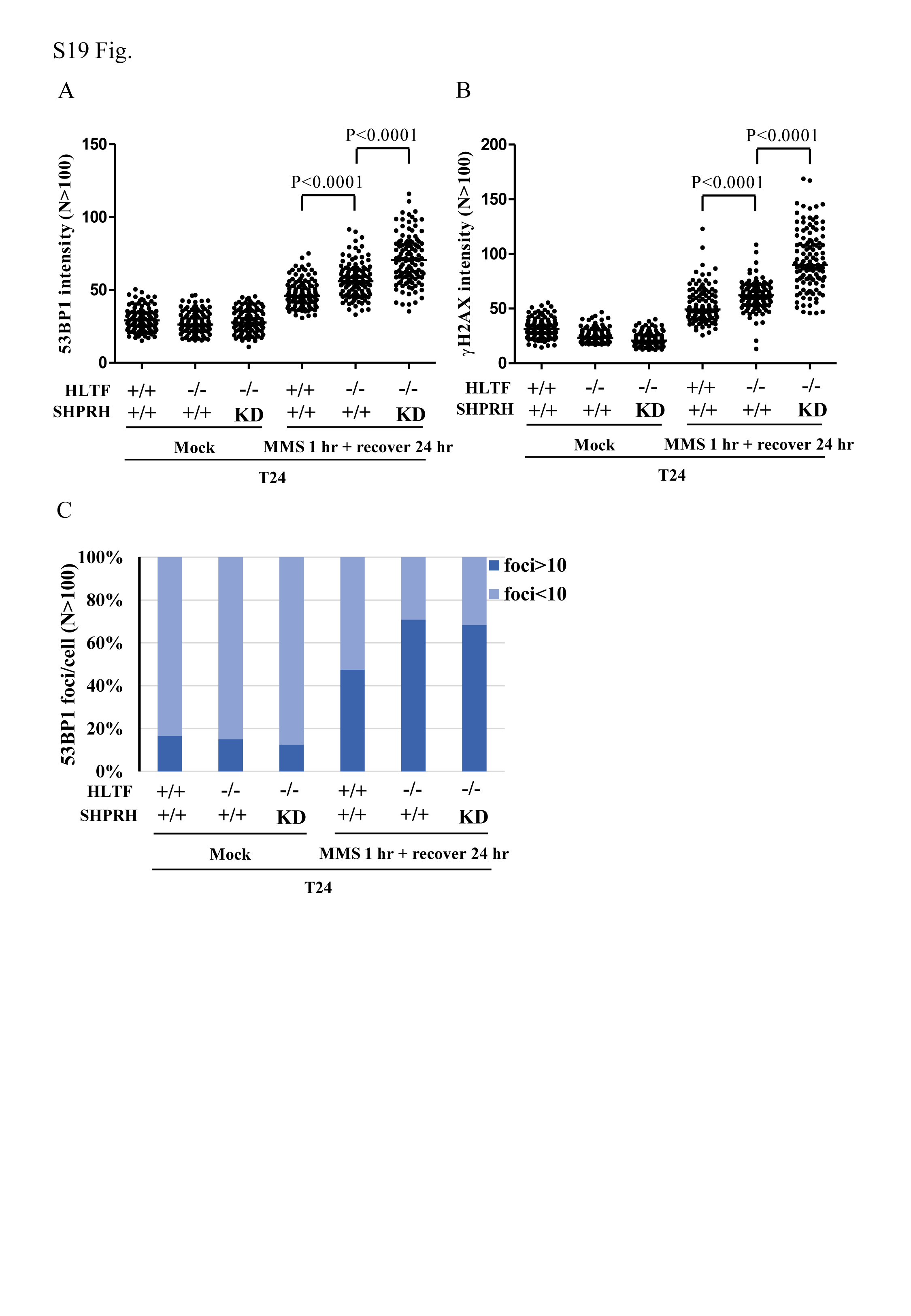

Supplement: S19 Fig — (A)(B) The intensity of 53BP1 and γH2AX in each cell was quantified using ZEN 3.3 software. Cells were treated with 0.01% MMS for 1 hour, followed by recovery in fresh medium for 24 hours. Cells were fixed and immunostained with 53BP1 and γH2AX antibodies. At least 100 cells from each cell line were quantified. (C) The number of 53BP1 foci from each cell was classified into two groups: <10 and >10, and the distributions of each group are indicated in the plot. (Raw 53BP1/γH2AX data in S16 Data). (TIF) [file pgen.1010545.s020.tif]

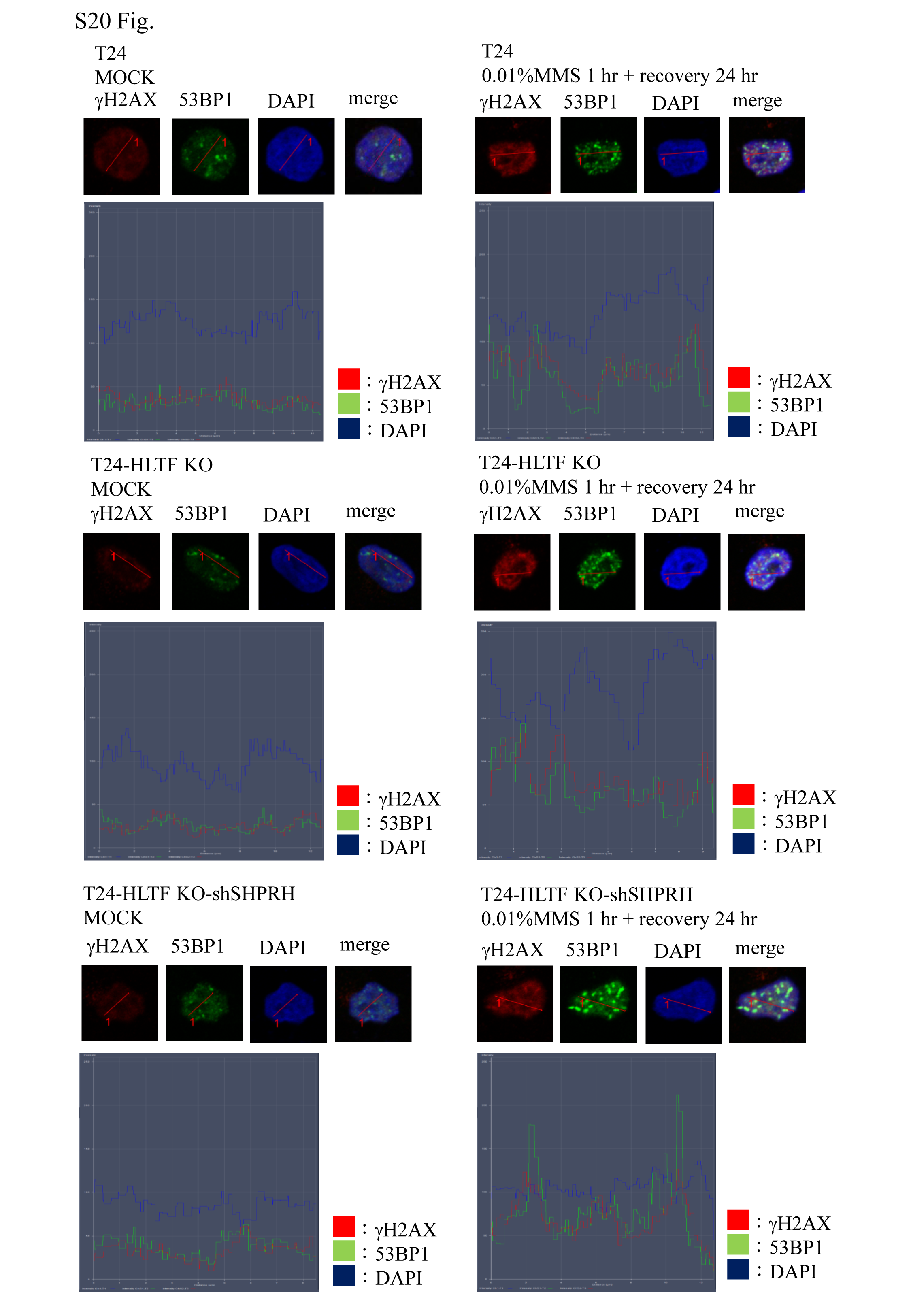

Supplement: S20 Fig — The confocal microscopy results of 53BP1 and γH2AX derived from each cell line. Cells were chronically treated with 0.01% MMS for 1 hr, followed by recovery in fresh medium for 24 hr. Cells were fixed and immunostained with 53BP1 and γH2AX antibodies. (TIF) [file pgen.1010545.s021.tif]
