# Supplementary material for: PARP1 recruits DNA translocases to restrain DNA replication and facilitate DNA repair
Source: PLoS Genet. 2022 Dec 13;18(12):e1010545. doi: 10.1371/journal.pgen.1010545 (PMC9794062; doi:10.1371/journal.pgen.1010545)
Supplement: S1 Table — (PDF) [file pgen.1010545.s022.pdf]

Table S1. The list of targeting sequences of shRNA used in this study.

Gene Symbol	Target sequence	Clone ID
LacZ	CGC GAT CGT AAT CAC CCG AGT	TRCN0000231722
PARP1	TTT GGT AAA GGG ATC TAT TTC	TRCN0000390913
HLTF	GCA GGT GGA GTT GGT TTG AAT	TRCN0000272618
SHPRH	ACG GAA CCA GAA GCG CTA TAT	TRCN0000235922
SMARCAL1	AGG TGT TGA TTG GGT ACA ATG	TRCN0000236116
SMARCAL1	CCA CAG TCC ACG TAG TCA AAT	TRCN0000236114
