# Supplementary material for: PARP1 recruits DNA translocases to restrain DNA replication and facilitate DNA repair
Source: PLoS Genet. 2022 Dec 13;18(12):e1010545. doi: 10.1371/journal.pgen.1010545 (PMC9794062; doi:10.1371/journal.pgen.1010545)
Supplement: S2 Table — (PDF) [file pgen.1010545.s023.pdf]

Table S2. The list of antibodies used in this study.

antibodies	WB Dilute concentration	PLA Dilute concentration	Catalog number	Source
Rabbit polyclonal anti-HLTF	1:5000	1:100	Cat#A300-230A	Bethyl Laboratories
Rabbit polyclonal anti-SHPRH	1:1000	1:100	ab80129	abcam
Rabbit polyclonal anti-ZRANB3	1:2000	1:100	Cat#A303-033A	Bethyl Laboratories
Rabbit polyclonal anti-ZRANB3	1:2000	1:50	PA5-65143	Invitrogen
Rabbit polyclonal anti-SMARCAL1	1:2000	1:100	Cat#A301-616A	Bethyl Laboratories
Mouse monoclonal anti-PARP1	1:2000	1:50	556494	BD Pharmingen
Rabbit polyclonal anti-phospho-Chk2	1:1000		#2661	cell signaling
Rabbit monoclonal anti-Chk2	1:500		#6334	cell signaling
Rabbit monoclonal anti-phospho-Chk1	1:1000		#2348	cell signaling
Mouse monoclonal anti-Chk1	1:500		sc-8408	Santa Cruz Biotechnology
Rabbit polyclonal anti-H2AX	1:2000		ab124781	abcam
Mouse monoclonal anti-γH2AX	1:2000		Cat#05-636	Millipore
Mouse monoclonal anti-α-tubulin	1:5000		GTX628802-01	Genetex
Rabbit polyclonal anti-53BP1			ab36823	abcam
Mouse monoclonal anti-biotin (PLA)		1:2000	Cat#200-002-211	Jackson ImmunoResearch
Rabbit polyclonal anti-biotin (PLA)		1:3000	Cat#A150-109A	Bethyl Laboratories
