# Supplementary material for: PARP1 recruits DNA translocases to restrain DNA replication and facilitate DNA repair
Source: PLoS Genet. 2022 Dec 13;18(12):e1010545. doi: 10.1371/journal.pgen.1010545 (PMC9794062; doi:10.1371/journal.pgen.1010545)
Supplement: Attachment — Submitted filename: response to referees v2.pdf [file pgen.1010545.s041.pdf]

National Cheng Kung University
Department of Life Sciences
No. 1, University Road,
Tainan City, 701
Taiwan
Phone: 886-6-2757575 ext 58100
FAX: 886-6-2742583

Hungjiun Liaw Ph.D.
Associate Professor
Phone: 886-6-2757575 ext 58127
email: liawh@mail.ncku.edu.tw

National Cheng Kung University
Dept of Life Sciences
No. 1 University Road
Tainan City 701, Taiwan

Oct. 31st, 2022

Dear Dr. Zha and Dr. McKinnon,

Thank you very much for your response and for the reviewer comments on our manuscript. We have revised the paper in response to the comments from the reviewers. Our point-by-point responses to those comments are included below.

Reviewer #1:

1. *Overall this referee finds the results indicating that PARP1 facilitates the recruitment of all 4 DNA translocases to damaged forks in a PAR-activity-dependent fashion shown in Fig 1, 2 and Fig. S1-6 are convincing and provided with all essential controls. Inhibiting PARP1 using Olaparib substantially reduced HLTF/biotin and SHPRH/biotin PLA foci, suggesting that PAR activity is critical for the recruitment of DNA translocases. However, besides inactivating PARP1, Olaparib also causes PARP1-DNA-bound complexes, which might interfere with replication complicating the interpretation.*

Olaparib treatment induces PARP1 trapping at the damage sites (1, 2). Further studies reveal that PARP1 is not trapped at the damage sites, but continuously recruited and exchanging at damages sites (3). It suggests that the recruitment of PARP1 to damaged forks is a dynamic process. In our study, we revealed that PARP1 activity is critical for the recruitment of DNA translocases to damaged forks. Similar to this phenomenon, the recruitment of XPC to damage sites is also regulated by PARP1 activity (4). We have to note that consistent with our results, several studies also showed that olaparib treatment increases replication speed and reduces the

frequency of reversed forks upon replication stress (5-8), compared to the mock treatment, indicating that PARP1 activity is critical for the formation of reversed forks. Since PARP1 activity also regulates chromatin compaction, the detailed mechanism of how PARP1 activity coordinates with DNA translocases requires further studies in the future.

2. *The mapping of the interaction domains in SHPRH in Fig 3b and supplementary Fig. S8, shows that the N-terminal part of SHPRH (aa 1-606), containing SNF2 and H15 domains, interacts with PARP1, and exclude the C-terminal part (aa 1090-1683). However, the middle part (aa 606-1090) has not been tested. Hence, this does not rule out the possibility that this part containing the PHD and SNF2 domains does also bind PARP1 and could harbor important functions. Is there a reason why the authors have not examined this portion of the protein? An interesting additional question is where does SHPRH bind to PARP1 and do the other DNA translocases bind to the same or a different part of PARP1?*

Previously, we identified that HLTF (1-763) containing the HIRAN and helicase ATP binding domains interacts with PARP1 (9). Since HLTF and SHPRH share structural and functional similarity, we focused on N-terminal domain of SHPRH.

Thanks for your suggestion. In order to map the overall interaction domains of SHPRH, we have generated the FLAG-tagged SHPRH (606-1090) containing the PHD domain and helicase ATP binding domain second part (HAB2) using in-fusion cloning (Supplementary Fig S9). In this revised manuscript, we corrected some mistakes of SHPRH construct map in the previous version and we changed “SNF2” domain to helicase ATP binding domain first part (HAB1) and second part (HAB2) based on UniProt Knowledgebase (UniProtKB) analysis. We found that SHPRH (1-605) containing the HAB1 and H15 domain and SHPRH (606-1090) containing the PHD and HAB2 domain interact with PARP1 (Fig 3b). We have to note that SHPRH (606-1090) has lower expression levels compared to the other construct. SHPRH (1090-1683) containing the RING domain and helicase C-terminal domain has marginal interaction with PARP1.

We further mapped the PARP1 domains that interact with each DNA translocase. We generated GFP-tagged PARP1, PARP1 (1-374) containing the DNA binding domain, PARP1 (356-476) containing the BRCT domain, and PARP1 (529-1014) containing the catalytic domain (Supplementary Fig S10a). We found that PARP1 (1-374) and PARP1 (356-532) interacts with HLTF, SHPRH, and SMARCAL1, but interacts with ZRANB3 weakly (Supplementary Fig S10b). Instead, ZRANB3 showed stronger interaction with PARP1 (529-1014). PARP1 (529-1014) also showed

weak and marginal interaction with SHPRH and SMARCAL1 (Supplementary Fig S10b). We conclude that PARP1 interacts with these DNA translocases through multiple contacts.

We have included these new data in page 10, line 11-26 and page 11, line 1-6, and Fig 3b and supplementary Fig S9 and S10a-c.

3. *It is remarkable that MMS did not enhance the in vivo interactions between PARP1 and all DNA translocases: HLTF, SHPRH, ZRANB3 and SMARCAL1, whereas one would expect that the interaction after DNA damage would most likely be strengthened, when the translocases are implicated in the response to replication stress. This is even more striking considering that MMS treatment induced an increase in the numbers of HLTF/PARP1, SHPRH/PARP1 and ZRANB3/PARP1 PLA foci (fig. 3c,d and S9). What is the explanation for this observation? Is this also the case for other types of replication-blocking DNA lesions such as UV-induced cyclobutane pyrimidine dimers, which require translesion synthesis for bypass?*

Based on our results, we think that PARP1 interacts with DNA translocases independent of DNA and PAR modification (Fig 3a)(9). However, PARP1 activity is critical for the recruitment of the PARP1/DNA translocases complex to damaged forks (Fig 2, supplementary Fig S4, S6, S7, S8). Since PARP1/DNA translocases complex is enriched at damaged forks upon MMS treatment, the numbers of HLTF/PARP1, SHPRH/PARP1 and ZRANB3/PARP1 PLA foci increase after MMS treatment (Fig 3c-d and Supplementary Fig S11). We speculate that PARP1 activity regulates chromatin compaction by recruiting ALC1 and APLF (10, 11) or by PARylating histones (12-14), which results in local chromatin structure change and allows the access of DNA translocases to chromatin. Consistent with this notion, HLTF is found in APLF and ALC1 complex in a large scale affinity purification mass spectrometry project (15). Both HLTF and SHPRH interact with PCNA (16). The HIRAN domain of HLTF interacts with 3' ssDNA (17). These multivalent interactions induce the recruitment of PARP1/DNA translocases complex to damaged forks. However, the detailed mechanism of how PARP1 activity coordinates the recruitment of DNA translocases to damage forks requires further investigation in the future.

To test whether UV treatment increases the numbers of HLTF/PARP1, SHPRH/PARP1, ZRANB3/PARP1, and SMARCAL1/PARP1 PLA foci, we performed the PLA assay. Human T24 cells were treated with 60 J/m^2 of UV irradiation to induce DNA damage. We found that UV treatment increased PARP1/HLTF, PARP1/SHPRH, PARP1/ZRANB3, and SMARCAL1/PARP1 PLA foci, similar to MMS treatment (Supplementary Fig S12).

We further tested whether UV induces the recruitment of DNA translocases to damaged forks, we performed the SIRF assay. Human T24 cells were treated with 60 J/m^2 of UV irradiation to induce DNA damage. We found that UV irradiation induced an increase of PLA foci of all four DNA translocases associated with damaged forks (Supplementary Fig S5a-c). Consistent with our results, a previous study also revealed that UV irradiation reduces fork progression and increases frequency of reversed forks using electron microscopy (5). Taken together, these results suggest that replication slowing and the formation of reversed forks are global responses to genotoxic treatments and this process requires PARP1/DNA translocases recruitment to damaged forks.

We have included these new data in page 8, line 26; page 9, line 1; page 11, line 16-18, and supplementary Fig S5 and Fig S12. We also put in discussion in page 16, line 27-28 and page 17, line 1-10.

4. *HLTF and SHPRH facilitate the loading of ZRANB3 to damaged replication forks in a PARP1-dependent fashion. Importantly, the authors demonstrate in two independent ways that SHPRH restrains DNA replication under conditions of HU-induced replication stress, providing the first evidence that this protein resembles its structurally-related cousin HLTF. Additionally, using different MMS concentration the investigators demonstrate that that HU, camptothecin, or MMS restrain DNA replication by the same mechanism, when similar DNA stress levels are induced, clarifying an apparent disparity in literature. The authors demonstrate that KO or depletion of PARP1, HLTF or SHPRH after MMS treatment activate Chk1 and Chk2 by phosphorylation (Fig. 6a) as well as γH2AX. Although overall the quality of immunoblots in the manuscript is high it appears that the γH2AX and the H2AX immunoblots are from different gels (See Fig. 6a). For proper comparison the same gel should be compared accompanied by the corresponding loading control (e.g., α-tubulin as used by the investigators), as done for p-Chk1 and Chk1 as well as pChk2 and Chk2. The same remark applies to Fig. 8b-d. Finally, the authors show that doubly depleted cells displayed elevated levels of 53BP1 foci (Fig. S14), suggesting that HLTF and SHPRH act synergistically to recover from MMS treatment.*

We have performed the western blotting using the same blot. Our results showed the similar trends as previous results. We have replaced these figures in Fig 6a and Fig 8b-e.

5. In the Discussion the authors state that their findings "… explain the mechanism of

PARP1 in fork progression restraint upon replication stress." This is somewhat overstated: although the results are interesting and provide further insight into the mechanisms of replication restrain still many questions remain and further investigation is needed before it is fully explained.

Thank you for your suggestion. We have removed the sentence from the manuscript.

Minor comment: The manuscript contains a number of grammatical errors.

We have sent our manuscript to NOVA Journal Experts for editing service, and corrected all grammatical errors.

Reviewer #2:

1. *We further identified the ability of SHPRH to restrain DNA replication upon replication stress, indicating that SHPRH is a DNA translocase. There is no direct evidence supporting this claim (see also below). It needs to be rephrased.*

Thank you for your suggestion. We have revised the sentence in abstract as follows, "We further identified the ability of SHPRH to restrain DNA replication upon replication stress, indicating that SHPRH itself could be a DNA translocase or a helper to facilitate DNA translocation. We have also included more explanation in page 12, line 18-22, as follows, "Currently, there is no evidence presented that SHPRH contains DNA translocase activity, which contributes to fork restraining. It requires further biochemical study to verify its DNA translocase activity. Alternatively, SHPRH could serve as a helper or scaffold protein to facilitate fork reversal. It awaits further studies in the future."

2. ***PARP1 deletion reduces DNA translocase levels at damaged forks***

Previous studies have shown that PARP1 depletion or treatment with the PARP1 inhibitor olaparib restrains DNA replication (25, 45-47). However, PARP1 has no helicase domains or DNA translocase activities, and the mechanism through which PARP1 restrains DNA replication is unknown.

There is a contradiction between these sentences, please rephrase, or specify the conditions! It is PARP1, not its depletion that restrains replication, as can be deduced from the second sentence and the cited literature.

We have rephrased our sentences as follows, "Previous studies have shown that

PARP1 restrains DNA replication. The depletion of PARP1 or treatment with the PARP1 inhibitor olaparib relieves replication restraint." We have revised these sentences in page 7, line 4-6.

3. *To test whether the PAR activity mediated by PARP1 facilitates DNA translocase recruitment, we introduced a PARP1-K893I mutant, which disrupts PAR activity (50, 51). We found that PARP1-K893I was not able to restore HLTF, SHPRH, or ZRANB3 PLA foci (Fig 2a-d and supplementary Fig. S6a-h). Cells treated with PARP1 inhibitor olaparib also showed reduced HLTF and SHPRH PLA foci after MMS treatment (supplementary Fig. 7a-f). Our results suggest that PARP1 facilitates the recruitment of DNA translocases to damaged forks in a PAR activity dependent manner.*

In a previous paper „The HLTF–PARP1 interaction in the progression and stability of damaged replication forks caused by methyl methanesulfonate" from the same group the conclusion was that the interaction between HLTF and PARP1 did not depend on PARylation, based on coIP experiments showing that the interaction did not increase upon or MMS treatment. In contrast, in similar experiments, MMS increased the interaction between PARP1 and BARD1. The authors should provide an explanation.

We found that DNA damage treatment did not enhance the interaction between PARP1 and DNA translocases (Fig 3a), and the interaction is not mediated through DNA (Fig 3a). Our in-silico analysis of the HLTF and SHPRH protein sequences did not reveal any PAR-binding motifs, macrodomains, PAR-binding zinc fingers (PBZ), WWE domains, or BRCT domains which are able to interact with PAR (18). Therefore, we think that PARP1 and DNA translocases form a complex and the interaction is not mediated through PAR modification. However, PAR activity is critical for the recruitment of the PARP1/DNA translocases complex to damaged forks (Fig 2, supplementary Fig S4, S6, S7, S8). We speculate that PARP1 activity regulates chromatin compaction by recruiting ALC1 and APLF (10, 11) or by PARylating histones (12-14), which results in local chromatin structure change and allows the access of DNA translocases to chromatin. Consistent with this notion, HLTF is found in APLF and ALC1 complex in a large scale affinity purification mass spectrometry project (15). Both HLTF and SHPRH interact with PCNA (16). The HIRAN domain of HLTF interacts with 3' ssDNA (17). These multivalent interactions induce the recruitment of PARP1/DNA translocases complex to damaged forks. However, the detailed mechanism of how PARP1 activity coordinates the recruitment of DNA translocases to damage forks requires further investigation in the future.

Previous studies have shown that PARP1 interacts with BARD1 through the interaction between PAR moiety and BRCT domain of BARD1(19, 20). Therefore, MMS treatment induces PARP1 autoPARylation and facilitates the recruitment of BARD1 to damage sites. Our previous results which MMS increased the interaction between PARP1 and BARD1 are consistent with these results (9).

4. *PARP1 associates with DNA translocases in vivo*

PARP1 was able to pull down all DNA translocases, HLTF, SHPRH, ZRANB3, and SMARCAL1 with HLTF showing the strongest interaction among these DNA translocases, and MMS treatment did not enhance these interactions. (Fig. 3a).

The coIP in Fig.3a is convincing only in the case of HLTF. With ZRANB3 a very weak signal can be observed. In the cases of SHPRH and SMARCAL1, the signal, if there is any, is extremely weak, a much better picture is needed to convince the reader about a positive interaction. Interestingly, with HLTF and ZRANB3, the signal is stronger in the mock-treated sample. This is in contrast to the model put forward in the manuscript, and also to the experimental results shown in Fig.3c where the number of detected PLA foci with these proteins and PARP1 increased upon MMS treatment. Does this mean that the complex containing PARP1 and these DNA translocases exists throughout the cell cycle independent of any treatments, and the treatment only affects their accumulation into foci at damaged forks? What is the authors' explanation?

Previously, our western blotting of coIP was performed using the same blot. After several stripping and reprobing, the signals for SHPRH, SMARCAL1, and ZRANB3 became weak. To improve the signal strength, we performed the anti-PARP1 coimmunoprecipitation with more cells (approximately $6x10^6$ cells), we were able to improve the signals. As shown in Fig 3a, all DNA translocases interact with PARP1 and the interaction is not mediated through DNA.

Indeed, based on our results, we think that PARP1 and DNA translocases form a complex independent of any treatment and PAR modifications. However, PAR activity is critical for the recruitment of the PARP1/DNA translocases complex to damaged forks. Similar to this phenomenon, the recruitment of XPC to damage sites is also regulated by PARP1 activity (4). We speculate that PARP1 activity regulates chromatin compaction by recruiting ALC1 and APLF (10, 11) or by PARylating histones (12-14), which results in local chromatin structure change and allows the access of DNA translocases to chromatin through multivalent interactions. In support of this notion, HLTF is found in APLF and ALC1 complex in a large scale affinity purification mass spectrometry project (15). Both HLTF and SHPRH interact with

PCNA (16). The HIRAN domain of HLTF interacts with 3' ssDNA (17). These multivalent interactions could facilitate the recruitment of PARP1/DNA translocases complex to damaged forks. However, the detailed mechanism of how PARP1 activity coordinates the recruitment of DNA translocases to damage forks requires further investigation in the future.

5. *We further mapped the interaction domains in SHPRH, which revealed that the N-terminal domain of SHPRH (aa 2-606), containing SNF2 and H15 domains, interacts with PARP1 (Fig 3b and supplementary Fig. S8).*
As coIP detects indirect and direct interactions, as well, the conclusion needs to be refined.

Indeed, coIP detects direct and indirect interactions. Therefore, we have revised these sentence as follows" Since the coimmunoprecipitation assay detects indirect and direct interactions between proteins, we cannot rule out the possibility that the interaction between PARP1 and SHPRH are mediated through other intermediates."

We have revised these sentences in page 10, line 20-23.

6. ***DNA translocases restrain DNA replication upon HU-induced replication stress***

SHPRH depletion also exhibited longer replication tracks upon HU treatment compared to wild type cells (Fig 5e). To the best of our knowledge, this represents the first evidence that SHPRH restrains DNA replication under conditions of replication stress.

After that sentence, it needs to be stated that there is no evidence presented that SHPRH contributes to fork restraining enzymatically. Theoretically, it could serve as a helper or scaffold protein, just like the DNA polymerase Rev1 does during DNA damage bypass.

Thanks for your suggestion. We have revised these sentence as follows, "Currently, there is no evidence presented that SHPRH contains DNA translocase activity, which contributes to fork restraining. It requires further biochemical study to verify its DNA translocase activity. Alternatively, SHPRH could serve as a helper or scaffold protein to facilitate fork reversal. It awaits further studies in the future."

We have revised these sentences in page 12, line 18-22.

7. ***Discussion***

We believed that these DNA translocases convert stalled forks into reversed forks to protect stalled fork from collapse and facilitate BER repair.

The recruited DNA translocases could initiate other repair pathways. Therefore, DNS repair instead of BER would be more appropriate.

Thanks for your suggestion. We have changed the sentence to "We believed that these DNA translocases convert stalled forks into reversed forks to protect stalled fork from collapse and facilitate DNA repair."

Thank you again. We look forward to hearing from you soon.

Sincerely yours,

Hungjiun Liaw, Ph.D.

References

1. Demin AA, Hirota K, Tsuda M, Adamowicz M, Hailstone R, Brazina J, et al. XRCC1 prevents toxic PARP1 trapping during DNA base excision repair. Mol Cell. 2021;81(14):3018-30.e5.

2. Murai J, Huang SY, Das BB, Renaud A, Zhang Y, Doroshow JH, et al. Trapping of PARP1 and PARP2 by Clinical PARP Inhibitors. Cancer Res. 2012;72(21):5588-99.

3. Shao Z, Lee BJ, Rouleau-Turcotte É, Langelier MF, Lin X, Estes VM, et al. Clinical PARP inhibitors do not abrogate PARP1 exchange at DNA damage sites in vivo. Nucleic Acids Res. 2020;48(17):9694-709.

4. Luijsterburg MS, Lindh M, Acs K, Vrouwe MG, Pines A, van Attikum H, et al. DDB2 promotes chromatin decondensation at UV-induced DNA damage. J Cell Biol. 2012;197(2):267-81.

5. Zellweger R, Dalcher D, Mutreja K, Berti M, Schmid JA, Herrador R, et al. Rad51-mediated replication fork reversal is a global response to genotoxic treatments in human cells. J Cell Biol. 2015;208(5):563-79.

6. Maya-Mendoza A, Moudry P, Merchut-Maya JM, Lee M, Strauss R, Bartek J. High speed of fork progression induces DNA replication stress and genomic instability. Nature. 2018;559(7713):279-84.

7. Berti M, Ray Chaudhuri A, Thangavel S, Gomathinayagam S, Kenig S, Vujanovic M, et al. Human RECQ1 promotes restart of replication forks reversed by DNA topoisomerase I inhibition. Nat Struct Mol Biol. 2013;20(3):347-54.

8. Sugimura K, Takebayashi S, Taguchi H, Takeda S, Okumura K. PARP-1 ensures regulation of replication fork progression by homologous recombination on damaged

DNA. J Cell Biol. 2008;183(7):1203-12.
9. Shiu JL, Wu CK, Chang SB, Sun YJ, Chen YJ, Lai CC, et al. The HLTF-PARP1 interaction in the progression and stability of damaged replication forks caused by methyl methanesulfonate. Oncogenesis. 2020;9(12):104.
10. Ahel D, Horejsi Z, Wiechens N, Polo SE, Garcia-Wilson E, Ahel I, et al. Poly(ADP-ribose)-dependent regulation of DNA repair by the chromatin remodeling enzyme ALC1. Science. 2009;325(5945):1240-3.
11. Ahel I, Ahel D, Matsusaka T, Clark AJ, Pines J, Boulton SJ, et al. Poly(ADP-ribose)-binding zinc finger motifs in DNA repair/checkpoint proteins. Nature. 2008;451(7174):81-5.
12. Messner S, Altmeyer M, Zhao H, Pozivil A, Roschitzki B, Gehrig P, et al. PARP1 ADP-ribosylates lysine residues of the core histone tails. Nucleic Acids Res. 2010;38(19):6350-62.
13. Poirier GG, de Murcia G, Jongstra-Bilen J, Niedergang C, Mandel P. Poly(ADP-ribosyl)ation of polynucleosomes causes relaxation of chromatin structure. Proc Natl Acad Sci U S A. 1982;79(11):3423-7.
14. Ray Chaudhuri A, Nussenzweig A. The multifaceted roles of PARP1 in DNA repair and chromatin remodelling. Nat Rev Mol Cell Biol. 2017;18(10):610-21.
15. Huttlin EL, Bruckner RJ, Navarrete-Perea J, Cannon JR, Baltier K, Gebreab F, et al. Dual proteome-scale networks reveal cell-specific remodeling of the human interactome. Cell. 2021;184(11):3022-40.e28.
16. Motegi A, Liaw HJ, Lee KY, Roest HP, Maas A, Wu X, et al. Polyubiquitination of proliferating cell nuclear antigen by HLTF and SHPRH prevents genomic instability from stalled replication forks. Proceedings of the National Academy of Sciences. 2008;105(34):12411-6.
17. Kile AC, Chavez DA, Bacal J, Eldirany S, Korzhnev DM, Bezsonova I, et al. HLTF's Ancient HIRAN Domain Binds 3' DNA Ends to Drive Replication Fork Reversal. Mol Cell. 2015;58(6):1090-100.
18. Gupte R, Liu Z, Kraus WL. PARPs and ADP-ribosylation: recent advances linking molecular functions to biological outcomes. Genes Dev. 2017;31(2):101-26.
19. Li M, Lu LY, Yang CY, Wang S, Yu X. The FHA and BRCT domains recognize ADP-ribosylation during DNA damage response. Genes Dev. 2013;27(16):1752-68.
20. Li M, Yu X. Function of BRCA1 in the DNA damage response is mediated by ADP-ribosylation. Cancer cell. 2013;23(5):693-704.
