# Supplementary material for: PARP1 recruits DNA translocases to restrain DNA replication and facilitate DNA repair
Source: PLoS Genet. 2022 Dec 13;18(12):e1010545. doi: 10.1371/journal.pgen.1010545 (PMC9794062; doi:10.1371/journal.pgen.1010545)
Supplement: Attachment — Submitted filename: 2022PlosGenPARP1review.docx [file pgen.1010545.s040.docx]

This manuscript investigates the connection between PARP1 and the DNA translocases HLTF, SHPRH, ZRANB3, and SMARCAL1. It builds on previous work from the same group (Shiu et al, 2020, Oncogenesis doi: 10.1038/s41389-020-00289-5) identifying an interaction between PARP1 and HLTF and investigating its role in the stabilization of damaged replication forks. Here, using almost the same high-tech methodology (SIRF, DNA fiber, immunofluorescence microscopy, etc) they extend their investigation to other DNA translocases. They generate knock-out cell lines to show that the translocases accumulate at damaged forks in a PARP1-dependent fashion, and the PARylation activity of PARP1 is necessary for that. They show interactions between PARP1 and the translocases by detecting the association of endogenous proteins (or also by overexpressing them in the case of SHPRH). They could detect increased co-localization of PARP1 and the translocases into foci after MMS treatment. Their results using low dose HU and MMS-treatment reveal that PARP1 and the translocases restrain replication forks without checkpoint activation, whereas high dose treatment results in checkpoint activation and reveals the role of these proteins in repair and replication restart. In summary, through PARP1 the translocases localize to damaged forks where they contribute to the stabilization, repair, and restart of damaged forks.

The results clarifying the role of DNA translocases and identifying new connections between these proteins having a substantial impact on genome stability can draw the attention of the broader scientific community. However, some of the conclusions are exaggerated without direct experimental evidence. Also, some experiments need to be improved so that the conclusions can be accepted.

Specific comments:

**Abstract**

*We further identified the ability of SHPRH to restrain DNA replication upon replication stress, indicating that SHPRH is a DNA translocase.*

There is no direct evidence supporting this claim (see also below). It needs to be rephrased.

**PARP1 deletion reduces DNA translocase levels at damaged forks**

*Previous studies have shown that PARP1 depletion or treatment with the PARP1 inhibitor olaparib restrains DNA replication (25, 45-47). However, PARP1 has no helicase domains or DNA translocase activities, and the mechanism through which PARP1 restrains DNA replication is unknown.*

There is a contradiction between these sentences, please rephrase, or specify the conditions! It is PARP1, not its depletion that restrains replication, as can be deduced from the second sentence and the cited literature.

*To test whether the PAR activity mediated by PARP1 facilitates DNA translocase recruitment, we introduced a PARP1-K893I mutant, which disrupts PAR activity (50, 51). We found that PARP1-K893I was not able to restore HLTF, SHPRH, or ZRANB3 PLA foci (Fig 2a-d and supplementary Fig. S6a-h). Cells treated with PARP1 inhibitor olaparib also showed reduced HLTF and SHPRH PLA foci after MMS treatment (supplementary Fig. 7a-f). Our results suggest that PARP1 facilitates the recruitment of DNA translocases to damaged forks in a PAR activity dependent manner.*

In a previous paper „*The HLTF–PARP1 interaction in the progression and stability of damaged replication forks caused by methyl methanesulfonate”* from the same group the conclusion was that the interaction between HLTF and PARP1 did not depend on PARylation, based on coIP experiments showing that the interaction did not increase upon or MMS treatment. In contrast, in similar experiments, MMS increased the interaction between PARP1 and BARD1. The authors should provide an explanation.

**PARP1 associates with DNA translocases *in vivo***

*PARP1 was able to pull down all DNA translocases, HLTF, SHPRH, ZRANB3, and SMARCAL1 with HLTF showing the strongest interaction among these DNA translocases, and MMS treatment did not enhance these interactions. (Fig. 3a).*

The coIP in Fig.3a is convincing only in the case of HLTF. With ZRANB3 a very weak signal can be observed. In the cases of SHPRH and SMARCAL1, the signal, if there is any, is extremely weak, a much better picture is needed to convince the reader about a positive interaction. Interestingly, with HLTF and ZRANB3, the signal is stronger in the mock-treated sample. This is in contrast to the model put forward in the manuscript, and also to the experimental results shown in Fig.3c where the number of detected PLA foci with these proteins and PARP1 increased upon MMS treatment. Does this mean that the complex containing PARP1 and these DNA translocases exists throughout the cell cycle independent of any treatments, and the treatment only affects their accumulation into foci at damaged forks? What is the authors’ explanation?

*We further mapped the interaction domains in SHPRH, which revealed that the N-terminal domain of SHPRH (aa 2-606), containing SNF2 and H15 domains, interacts with PARP1 (Fig 3b and supplementary Fig. S8)*.

As coIP detects indirect and direct interactions, as well, the conclusion needs to be refined.

**DNA translocases restrain DNA replication upon HU-induced replication stress**

*SHPRH depletion also exhibited longer replication tracks upon HU treatment compared to wild type cells (Fig 5e). To the best of our knowledge, this represents the first evidence that SHPRH restrains DNA replication under conditions of replication stress.*

After that sentence, it needs to be stated that there is no evidence presented that SHPRH contributes to fork restraining enzymatically. Theoretically, it could serve as a helper or scaffold protein, just like the DNA polymerase Rev1 does during DNA damage bypass.

**Discussion**

*We believed that these DNA translocases convert stalled forks into reversed forks to protect stalled fork from collapse and facilitate BER repair.*

The recruited DNA translocases could initiate other repair pathways. Therefore, DNS repair instead of BER would be more appropriate.
